# Iono–Magnonic Reservoir Computing With Chaotic Spin Wave Interference Manipulated by Ion‐Gating

**DOI:** 10.1002/advs.202411777

**Published:** 2024-11-17

**Authors:** Wataru Namiki, Daiki Nishioka, Yuki Nomura, Takashi Tsuchiya, Kazuo Yamamoto, Kazuya Terabe

**Affiliations:** ^1^ Research Center for Materials Nanoarchitectonics (MANA) National Institute for Materials Science 1‐1 Namiki Tsukuba Ibaraki 305‐0044 Japan; ^2^ Faculty of Science Tokyo University of Science 6‐3‐1 Niijuku Katsushika Tokyo 125‐8585 Japan; ^3^ Nanostructures Research Laboratory Japan Fine Ceramics Center 2‐4‐1 Mutsuno, Atsuta Nagoya Aichi 456‐8587 Japan

**Keywords:** nonlinear interference, proton, redox, reservoir computing, solid‐state electrolyte, spin wave

## Abstract

Physical reservoirs are a promising approach for realizing high‐performance artificial intelligence devices utilizing physical devices. Although nonlinear interfered spin‐wave multi‐detection exhibits high nonlinearity and the ability to map in high dimensional feature space, it does not have sufficient performance to process time‐series data precisely. Herein, development of an iono–magnonic reservoir by combining such interfered spin wave multi‐detection and ion‐gating involving protonation‐induced redox reaction triggered by the application of voltage is reported. This study is the first to report the manipulation of the propagating spin wave property by ion‐gating and the application of the same to physical reservoir computing. The subject iono–magnonic reservoir can generate various reservoir states in a single homogenous medium by utilizing a spin wave property modulated by ion‐gating. Utilizing the strong nonlinearity resulting from chaos, the reservoir shows good computational performance in completing the Mackey–Glass chaotic time‐series prediction task, and the performance is comparable to that exhibited by simulated neural networks.

## Graphical abstract

This work presents an iono–magnonic reservoir computing device that combines interfered spin wave multi‐detection and ion‐gating involving a protonation‐induced redox reaction. The iono–magnonic reservoir can generate various reservoir states by utilizing spin wave properties modulated by the ion‐gating. With the strong nonlinearity resulting from chaos, the reservoir performs the chaotic time‐series data prediction task with excellent computational performance.

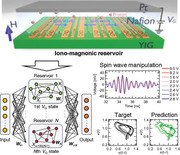

## 1 Introduction

Physical reservoir computing is attracting attention as a promising method for implementing high‐efficiency artificial intelligence, which is due to the system having fewer learning parameters than deep neural networks with weight parameters above 10^11^, and its ability to mimic biological systems with nonbiological elements.^[^ ^1^, ^2^, ^3^, ^4^, ^5^, ^6^ ^]^ These advantages can lead to the utilization of various physical devices (e.g., electrical circuits,^[^ ^7^, ^8^, ^9^ ^]^ electrochemical elements,^[^ ^10^, ^11^, ^12^, ^13^, ^14^ ^]^ magnetic devices,^[^ ^15^, ^16^, ^17^, ^18^, ^19^, ^20^, ^21^, ^22^, ^23^, ^24^, ^25^ ^]^ optical elements,^[^ ^26^, ^27^, ^28^, ^29^, ^30^ ^]^ robotic system,^[^ ^31^ ^]^ ion‐gating devices,^[^ ^32^, ^33^, ^34^, ^35^ ^]^ and so on^[^ ^36^, ^37^ ^]^), with three features: nonlinear transformation, short‐term memory, and the ability to map in higher dimensional feature space. However, some issues remain on the road to the successful implementation of practical physical reservoir computing, these being high electrical power consumption, low accuracy rates, and the large volumes associated with such systems.

In our previous work, we found that nonlinear interfered spin wave multi‐detection exhibits the lowest errors in performing the second‐order nonlinear dynamic equation task (normalized mean square error [NMSE]: 8.37 × 10^−5^) and second‐order nonlinear autoregressive moving average (NARMA2) task (NMSE using variance [NMSE_var._]: 1.81 × 10^−2^) in reported micro–nano physical reservoir systems, which is due to its strong nonlinearity and its elevated ability to map in higher dimensions.^[^ ^21^ ^]^ Although the performance of the physical reservoir may be improved by an incremental increase in the number of detectors or by utilizing various waveguides, these changes lead to fatal issues that worsen the degree of integration due to expansion of the reservoir volume, the increased amount of wiring required, and so on. To overcome such problems, in situ manipulation of spin wave property in a single homogenous medium is required. This scheme connects to advantage, which is the improvement of the ability to map in higher dimensional feature space without increasing the number of physical devices.

Herein, we report on our fabrication of an iono–magnonic reservoir device that will allow the fields of ionics and magnonics to collaborate to improve reservoir performance. This approach can be achieved by ion‐gating, which can significantly manipulate magnetic properties (i.e., saturation magnetization and magnetic anisotropy) through redox reactions triggered by the application of gate voltage (*V*_G_)^[^ ^38^, ^39^, ^40^ ^]^ because the magnetic property closely relates to spin wave properties. This study is the first study of the manipulation of chaotic spin wave interference as an information carrier achieved with a solid‐state electrolyte and its application to high‐performance reservoir computing.^[^ ^41^ ^]^ The NMSE and NMSE_var._ of the second‐order nonlinear dynamic equation task and NARMA2 are 6.41 × 10^−5^ and 9.53 × 10^−3^, corresponding to 76.6% and 52.7% of the NMSE and NMSE_var._ of the nonlinear interfered spin wave multi‐detection.^[^ ^21^ ^]^ This drastic improvement results from the excellent nonlinearity of chaotic spin wave interference and the ability of ion‐gating to map in higher dimensional space, which allows the iono–magnonic reservoir to achieve the good performance, compared to other physical reservoirs thus far reported.

## 2 Spin Wave Manipulation With an Iono–Magnonic Device

**Figure 1:**
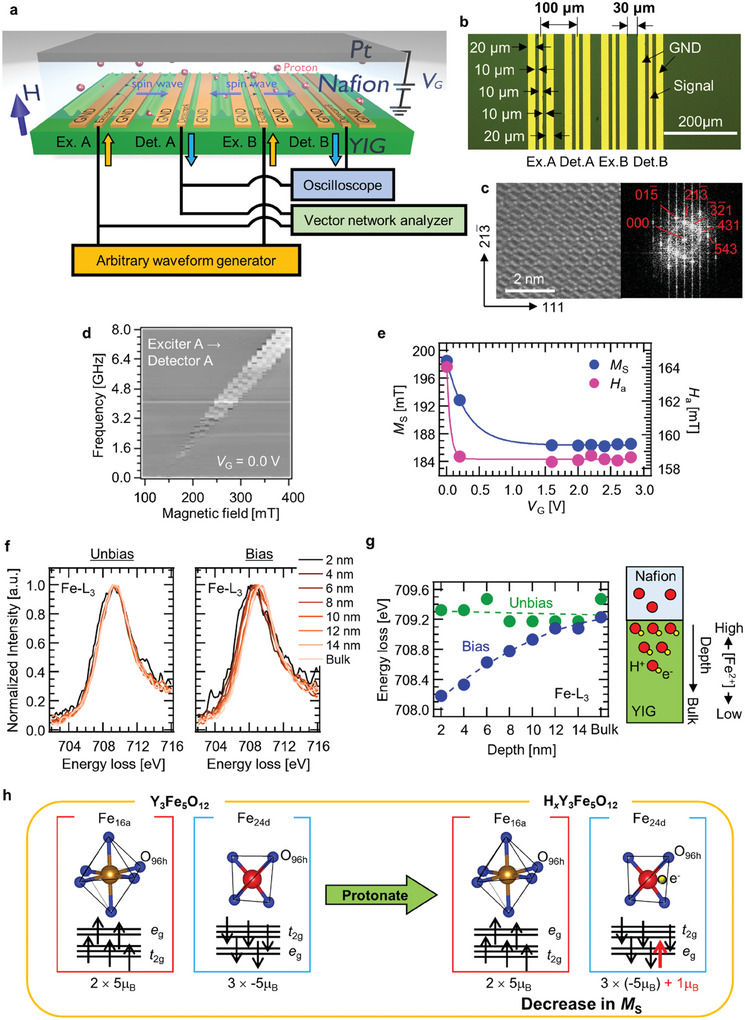
Magnetic property manipulation through electronic carrier tuning by ion‐gating. a) Iono–magnonic with Y_3_Fe_5_O_12_ (YIG) single crystal and Nafion and its experimental configuration. b) Optical microscope image of antennas deposited on the single crystal. c) Scanning transmission electron microscope image of the single crystal and its fast Fourier transform image. d) Spin wave spectroscopy of de‐embedded transmission signal, measured at gate voltage *V*_G_ of 0.0 V. e) Saturated magnetization *M*_S_ and anisotropic field *H*_a_ as a function of *V*_G_. f) Electron energy‐loss spectra variation of Fe–L_3_ of “Unbias” and “Bias”. g) Energy‐loss variation at various depths from the YIG/Nafion interface. h) Schematic illustration of spin configuration manipulated by ion‐gating.

**Figure** **1**a is a schematic illustration of an iono–magnonic, consisting of Y_3_Fe_5_O_12_ (YIG) single crystal and a Nafion electrolyte and its measurement configuration. The device had two exciters and two detectors, consisting of coplanar waveguides with signal and ground (GND) lines with widths of 10 and 20 µm, respectively. The detailed waveguide dimensions of the device are shown in Figure 1b,c, comprising a scanning transmission electron microscopy (STEM) image and the fast Fourier transform of a cross‐section of the YIG region. These images show that the YIG was highly crystalline. A Nafion, proton conductive solid‐state electrolyte, with a Pt gate electrode, was attached to the YIG. A *V*_G_ ranging from 0.0 to 2.8 V was applied between Pt thin film and GND. Then, the electronic carrier was doped in the YIG by the protonation‐induced redox reaction of the YIG, triggered by the *V*_G_ application.^[^ ^40^ ^]^ Details of the iono–magnonic and its experimental set‐up are described in the Experimental Section. To investigate the spin wave property variation at various *V*_G_, the magnetic field dependence of the spin wave frequency was measured, as shown in the *V*_G_ = 0.0 V example in Figure 1d. Frequency *f* of the forward volume magnetostatic wave (FVMSW) mode is described as follows:

(1) $$f=\gamma \sqrt{\left(H-H_{a}\right)\left\{\left(H-H_{a}\right)+M_{S}\left(1-\frac{1-e^{-kd}}{kd}\right)\right\}}$$

where *γ*, *H*, *H*_a_, *M*_S_, *k*, and *d* are the gyromagnetic ratio, applied external field, magnetic anisotropy field, saturation magnetization, wave number of the spin wave, and YIG thickness, respectively. The *γ* was set to 28 GHz mT^−1^.^[^ ^21^, ^42^ ^]^ The *V*_G_ dependence of *M*_S_ and *H*_a_ were obtained from the fitting result using Equation (1), as shown in Figure 1e. The *M*_S_ of 198.5 mT at *V*_G_ = 0.0 V was in good agreement with an *M*_S_ of 198.4 mT obtained from the magnetization measurement in a pristine YIG single crystal. The *H*_a_ at *V*_G_ = 0.0 V was 158.7 mT. Increasing *V*_G_, corresponding to electron doping, decreased both *M*_S_ and *H*_a_. The change saturated at the region of *V*_G_ ≥ 1.6 V. Details of the magnetic properties of the iono–magnonic are shown in S1, Supporting Information.^[^ ^21^, ^42^ ^]^

Such significant magnetic property variation results from the manipulation of electronic structure.^[^ ^38^, ^39^, ^40^ ^]^ To investigate the change of valence state by protonation, electron energy‐loss (EEL) spectroscopy was carried out on two YIG single crystals. One was an unbiased YIG with no history of voltage application (“Unbias”), and the other was a biased YIG with a history of voltage application of 2.8 V (“Bias”). Figure 1f shows the depth dependence of the EEL spectra of Fe‐L_3_. In the “Unbias” spectra, there was no significant chemical shift of the Fe–L_3_ and Fe ion existed as trivalent in the YIG.^[^ ^43^ ^]^ On the other hand, in the “Bias” spectra, the peak shifted to the lower energy side as the measurement position approached the interference from the bulk region due to electrochemical reduction.^[^ ^40^ ^]^ As shown in Figure 1g, the depth dependence of the peak position of “Bias” was obviously observed. The peak position at the bulk region was in good agreement with that of “Unbias”, indicating that Fe ions near the Nafion/YIG interface were electrochemically reduced from trivalent to divalent states. In contrast, the Fe ions remained trivalent in bulk. This result shows that the effect of protonation is strongest at a thin region, ≈10 nm thick near the interface and due to the short diffusion length of the proton, gradually weakened as the depth increased. The electrochemical reaction that occurred is described as follows:

(2) $$Y_{3}Fe_{5}O_{12}+xH^{+}+xe^{-}\to H_{x}Y_{3}Fe_{5}O_{12}$$

and

(3) $$5Fe^{3+}+xe^{-}\to \left(5-x\right)Fe^{3+}+xFe^{2+}$$

As the Fe ions in YIG determine its magnetic property,^[^ ^43^ ^]^ it is expected that magnetization and magnetic anisotropy are changed by any reduction of Fe^3+^. From the changes in magnetic properties and from EEL spectroscopy, change of the spin configuration in Fe ions can be assumed, as shown in Figure 1h. YIG has two Fe ion sites; one is Fe_16a_, which is found at the center of an octahedron, surrounded by six oxygen ions (O_96h_) that occupy its six corners, and the d orbitals of which are half‐filled by up spins. The second is Fe24d, which is located at the center of a tetrahedron, surrounded by four O_96h_ ions that occupy its four corners, and the d orbitals of which are half‐filled by down spins.^[^ ^44^ ^]^ Although the Fe ions in YIG have a magnetic moment of 5µ_B_, effective *M*_S_ is determined by the magnetic moment in Fe_24d_ due to the occupancy ratio of Fe_16a_:Fe_24d_ = 2:3 because YIG is a ferrimagnetic material. Thus, the doped electron occupies an orbit as up spin in Fe_24d_, which is deduced from the experimental result revealing a decrease in *M*_S_.

## 3 Manipulated Spin Wave Property and Nonlinear Interference

To investigate the effect of electronic structure manipulation on spin dynamics, we measured spin wave property variation at various *V*_G_. The spin wave spectra acquired at a magnetic field of 170 mT are shown in **Figure** **2**a. The peak, denoted by a solid black arrow, shifted to the higher frequency sides as *V*_G_ increased, which indicated an increase in spin wave frequency. Frequency variation at various *V*_G_ is summarized in Figure 2b. The frequency increased from ≈1.30 to 1.32 GHz, corresponding to a shift of 20.5 MHz, and these increase ratios were 1.58%. Similar shifts of three significant peaks were observed in the spin wave spectra acquired at a magnetic field of 190 mT, as shown in Figure 2c. These peaks were the result of signals from spin waves with different wavenumbers. The wavenumber of a spin wave excited by the antenna was determined by the geometry of the excitation antenna. This was because spin waves were excited by the coupling of the spin waves with the induced magnetic field that resulted from the RF current pulse flowing through the antenna. As mentioned in our previous work, in this study, the three main wavenumbers of the current flowing through the coplanar antenna used were 0.118, 0.311, and 0.492 µm^−1^, and three spin waves with these wavenumbers propagated in the YIG.^[^ ^21^ ^]^ In the spin wave frequency spectrum at 190 mT, three spin waves with wavenumbers of 0.118, 0.311, and 0.492 µm^−1^ corresponded to the three peaks at 1.53, 1.79, and 2.04 GHz, respectively. The frequency shift of the spin wave at 0.118 µm^−1^ was 21.5 MHz, while that of the spin wave at 0.311 µm^−1^ was 24.2 MHz and that of the spin wave at 0.492 µm^−1^ was 11.9 MHz. Although the frequency shift of the spin wave at 0.492 µm^−1^ was relatively small, the trend for increased frequency with respect to *V*_G_ was consistent. Spin wave packets were changed by applied *V*_G_ variation, as shown in Figure 2g. There was a finite difference between spin wave packets at certain *V*_G_ and *V*_G_ = 0.0 V. Relatively small differences (significant differences with waveform shape) were observed at *V*_G_ ≤ 2.0 V (*V*_G_ ≥ 2.2 V). This was due to the frequency shift resulting from the *V*_G_ application, which was shown in Figure 2b,d–f. In addition, the amplitude normalized at *V*_G_ = 0.0 V of spin waves was varied by *V*_G_ application, as shown in Figure 2h. Thus, it was found that the *V*_G_ application could largely manipulate the spin wave property through the electronic structure. Here, although the conditions under the magnetic fields of 169 and 186 mT were different from the conditions of the spin wave resonance spectra, the *V*_G_ dependence of the spin wave frequency at the magnetic field ranged from 170 to 190 mT, closest to the 169 and 186 mT, which could examine the contribution of *V*_G_ application to the manipulation of the spin wave properties.

Figure 2Spin wave property manipulation and improvement of reservoir performance. a) De‐embedded S_21_ spectrum variation at various *V*_G_, measured at a magnetic field of 170 mT. A spin wave propagating from Exciter A to Detector A is acquired. b) Frequency variation at various *V*_G_. The frequency is represented by the solid black arrow described in (a). c) De‐embedded S_21_ spectrum variation at various *V*_G_, measured at a magnetic field of 190 mT. d–f) Frequency variation at various *V*_G_. The frequency is represented by (d–f) denoted in (c), respectively. g) (Upper panel) *V*_G_ dependence of spin wave signal transmitted from Exciter A to Detector A at time domain. (Lower panel) Difference between spin wave signal at *V*_G_ and spin wave signal at *V*_G_ = 0.0 V. h) *V*_G_ dependence of normalized output amplitude of spin wave calculated from Figure 2e. The filled blue and green circles represent amplitudes of 169 and 186 mT, respectively. The dashed line represents the guidelines. i) Interfered spin wave signal at Detector A, in a magnetic field of 186 mT. j,k) Cropped signals of (i). l) Interfered spin wave signal at Detector B, in a magnetic field of 186 mT. m,n) Cropped signals of (l). o) Schematic illustration of the difference between reservoir performance in cases without interference, multi‐detection, and *V*_G_ (left panel) and with interference, multi‐detection, and *V*_G_ (right panel).

**Figure:**
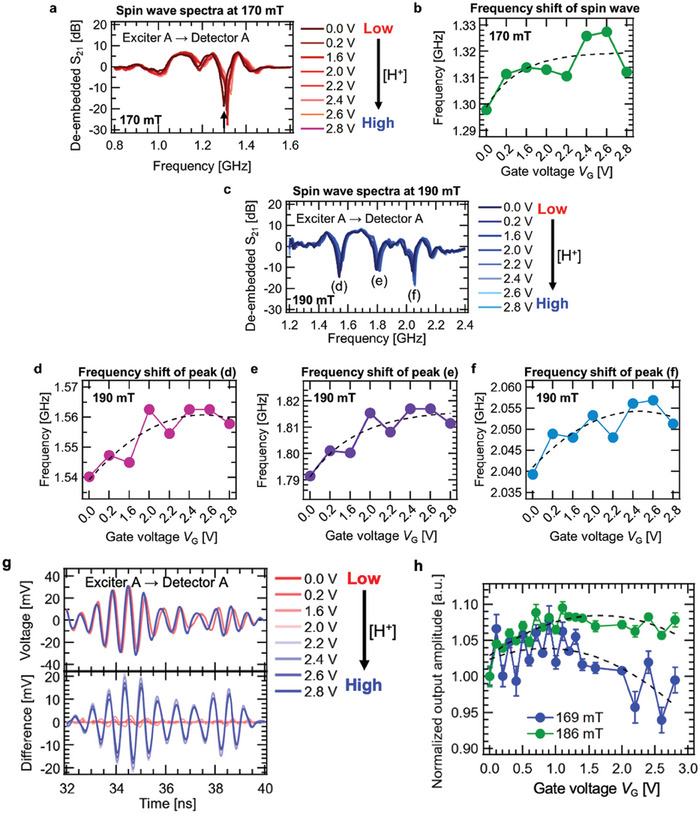

**Figure:**
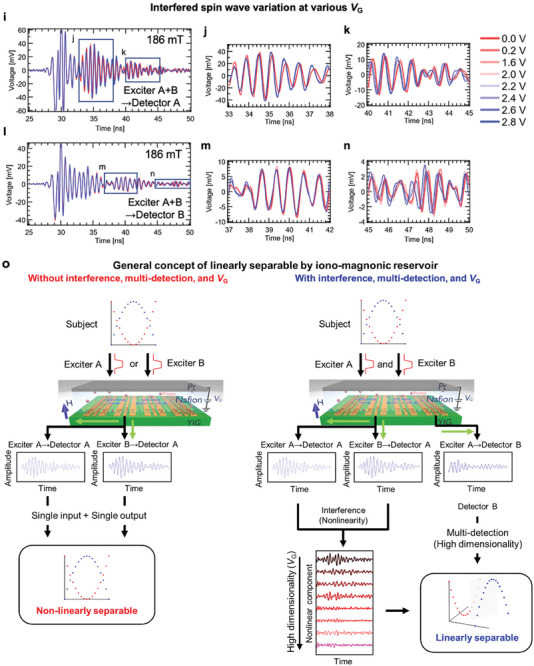

Figure 2i–k shows interfered spin wave variation at various *V*_G_, detected at Detector A. Large frequency variations are observed for the entire time domain due to spin wave property manipulated by *V*_G_ application, as shown in Figure 2b,d,f. Similar variations are also shown in the spin wave signal detected at Detector B, as shown in Figure 2l–n. Detector B detects not only the spin wave excited at Exciter B, but also the spin wave excited at Exciter A. The spin wave generated at Exciter B propagates, while the spin wave excited at one discrete time *k* is affected by the precession of the spin wave excited at the previous *k*. This is not interference of waves facing each other, but the behavior of waves affected by short‐term memory. The nonlinear interfered spin wave, as detected at Detector A, passes directly through Excitor B and arrives at Detector B. At such time, the spin wave propagates under the influence of the spin wave at Exciter B, which propagates at the previous *k*, and the residual spin wave at Exciter A. In other words, a wave strongly affected by nonlinearity and short‐term memory arrives at Detector B and imparts reservoir nonlinearity and short‐term memory. The amplitude at both magnetic fields of 169 and 186 mT decreases as *V*_G_ increases. This is because nonlinear spin wave interference results from the dipole–dipole interaction between spin precessions; the interaction becomes weaker due to a decrease in the spin magnetic moment of Fe ion as a result of electrochemical reduction, as shown in Figure 1h;^[^ ^19^, ^45^ ^]^ and the *V*_G_ application leads to modulation of the degree of nonlinearity of the interfered spin wave. Note that the effect of electron doping has a depth gradient, and the change in electronic structure shown in Figure 1h is heterogeneous. However, the doping heterogeneity resulting from proton insertion does not directly induce nonlinearity to the iono–magnonic reservoir but rather modulates the nonlinearity resulting from spin wave interference. Spin waves propagate both in regions where the magnetic properties have changed, and in regions where they have not. The spin waves detected by a vector network analyzer or oscilloscope synthesize the spin waves propagating in these regions (or the interaction between the spin waves propagating in the two regions). As shown in S2, Supporting Information, the nonlinear component of the interference decreases as *V*_G_ increases. The intensity of the saturation magnetization also decreases, suggesting that *V*_G_ weakens the dipole interaction between spin waves and thus weakens the nonlinear interference. Figure 2o schematically shows how the performance of the reservoir, which enables the linear separation of non‐linearly separable data by nonlinearly mapping them to higher dimensional space, is dramatically improved by adding in situ manipulation of the spin wave property by *V*_G_, in addition to spin wave interference and multi‐detection. A comparison with voltage control of magnetic properties is also given in S2, Supporting Information.^[^ ^46^, ^47^ ^]^ A general spin wave reservoir without interference, multi‐detection, and *V*_G_ has poor nonlinearity and low dimensionality, and such reservoir cannot solve given complex tasks (“non‐linearly separatable”). On the other hand, as spin waves with various nonlinearity are generated depending on the amplitude of the *V*_G_, it is expected that the interfered spin wave manipulation achieved with the iono–magnonic increases the dimension of the feature space mapped by the reservoir, in addition to the nonlinearity, compared with a conventional spin wave reservoir, as shown in the right‐hand panel of Figure 2o. The colored waveforms connected by arrows extending from the antenna represent the signals to be detected. The spin wave properties in each voltage state are different in amplitude and frequency and, as such, have different short‐term memory and nonlinearity due to spin wave interference. In the right‐hand panel, eight colored waveforms show that the interference between the two signals has a strong nonlinear component, which the *V*_G_ can control. Thus, the iono–magnonic significantly strengthens the role of the reservoir in making input time‐series data linearly separatable by mapping nonlinearly separatable information to a higher dimensional space (“linearly separatable”), and such a reservoir can solve given tasks.^[^ ^5^ ^]^

## 4 Performance Evaluation of the Iono–Magnonic Reservoir

**Figure 3:**
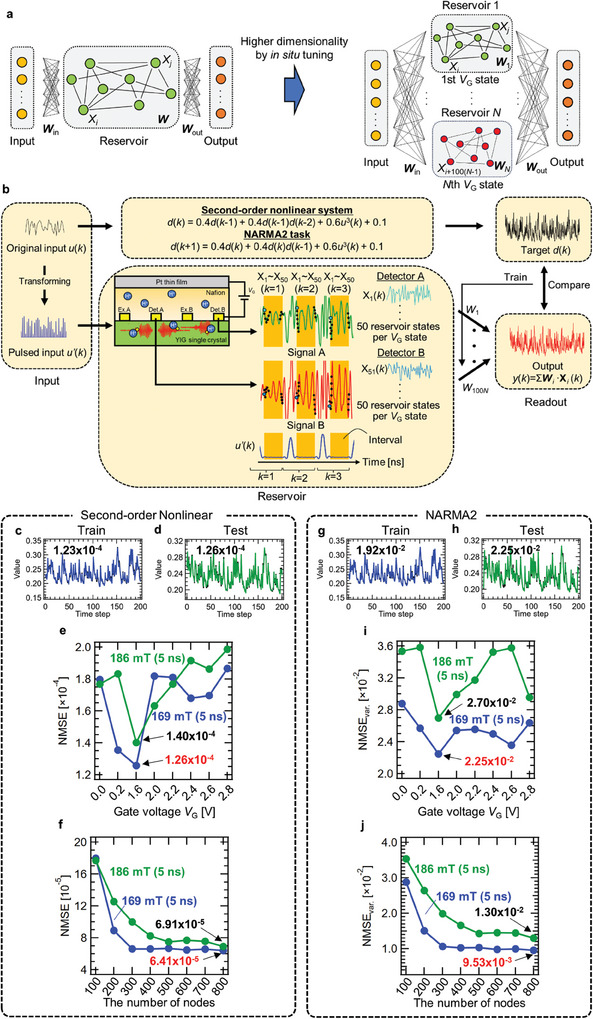
Concept of a reservoir computing system with an iono–magnonic reservoir for a time‐series data processing task. a) General concepts of a typical reservoir computing system (left‐hand panel) and of reservoir computing with multiple reservoirs achieved with in situ tuning (right‐hand panel). *N V*_G_ states produce *N* reservoir layers. b) Process flow diagram of time‐series processing tasks using a reservoir computing system with an iono–magnonic reservoir. Fifty reservoir states are obtained from a detector at a *V*_G_ state. The yellow‐green dot and the purple dot denote extracted *X*_1_ and *X*_51_, respectively. Similarly, by extracting the values for *X*_2_–*X*_50_ and *X*_52_–*X*_100_ represented as the black circles, a total of 100 waveforms (i.e., 50 reservoir states/Detector A + 50 reservoir states/Detector B) are generated. The waveforms for *X*_2_–*X*_50_ and *X*_52_–*X*_100_ are omitted from the figure. c,d) Predicted results at the training phase (c) and testing phase (d) of a second‐order nonlinear dynamical equation task. The black, blue, and green lines represent the target and output waveforms at the training and testing phases, respectively. e,f) NMSE variations at various gate voltages *V*_G_ (e) and the number of nodes variation (f). g,h) Predicted results at the training phase (g) and testing phase (h) of the NARMA2 task. The black, blue, and green lines represent the target and output waveforms at the training and testing phases, respectively. i,j) NMSE_var._ variations at various gate voltages *V*_G_ (i) and the number of nodes variation (j).

**Figure** **3**a shows the general concept of a reservoir computing model, which consists of three layers: input, reservoir, and readout. Time‐series data is input into the device and transformed nonlinearly into waveforms referred to as reservoir states, at time step domain by *n* virtual nodes (*X*_1_ ≈ *X_n_*) in reservoir layers.^[^ ^7^ ^]^ Then, the reservoir states are crossed by *n* weight parameters (≈*W*_1_–*W_n_*), which connect the reservoir layer to the readout layer so as to generate reservoir output *y* in the readout layer.^[^ ^5^ ^]^ Here, the performance of the overall reservoir network closely relates to the characteristics of the reservoir based on physical dynamics. Thus, we construct a network with reservoir layers connected in parallel through the utilization of the spin wave property by ion‐gating and enhance the expressive power of the network to improve the processing performance of the physical reservoir.

The process flow for the tasks necessary to verify the performance of the iono–magnonic reservoir is shown in Figure 3b. The original input *u*(*k*) and pulsed input *u*′(*k*) had a total length of *k* = 5000. Here, *k* denotes a discrete time. Time steps were separated into training phases with a time step of 3500 and test phases with a time step of 500 after the first‐time step of 1000 was discarded. There were two detectors (Detector A and Detector B) on the YIG, and 50 nodes were extracted per detector. As shown in Figure 3b, the original random wave was converted into a pulsed waveform. However, no masking was performed to change the input amplitude in order to improve the ability of the reservoir. 50 nodes were extracted per pulse interval of the random wave (i.e., a discrete time *k*). As these 50 nodes were extracted per detector and there were two detectors, 100 nodes per voltage condition could be extracted from the period corresponding to the interval. As the maximum number of voltage conditions *N* is 8, the number of nodes that could be used was 100 *N*. Thus, the maximum number was 800 nodes (= 100 nodes/*V*_G_ state × 8 *V*_G_ states). The weights (i.e., ***W***) in the reservoir layer depicted in Figure 3a are the coupling strengths between virtual nodes in the time direction. *W*_s_ between neighboring virtual nodes (e.g., *X*_1_ and *X*_2_) was strong, while weights between distant virtual nodes (e.g., *X*_1_ and *X*_50_) were weak. In addition, the coupling between neighboring virtual nodes became weaker when the pulse interval was made sufficiently long. It is difficult here to calculate the ***W*** between nodes in the reservoir from the current spin wave output signal. Solving second‐order nonlinear system and NARMA2 task was widely performed.^[^ ^5^, ^8^, ^9^, ^10^, ^11^, ^12^, ^16^, ^17^, ^21^, ^31^, ^32^, ^33^, ^34^, ^35^, ^37^, ^48^ ^]^ Details of these tasks are described in the Experimental Section. Figure 3c,d shows comparative results in the training and test phases of the second‐order nonlinear dynamic equation task, in the condition of magnetic field 169 mT and pulse interval 5 ns. The NMSE at the training phase was 1.23 × 10^−4^. The comparative results in the testing phase are shown in Figure 3d. NMSE for the testing phase exhibited a similar value of 1.26 × 10^−4^. These two waveforms generated by the iono–magnonic reservoir were in good agreement with target waveforms. NMSE variation at various *V*_G_ applications is shown in Figure 3e. Although NMSE is changed by *V*_G_ application, the minimal NMSE is achieved at *V*_G_ = 1.6 V with a magnetic field of 169 mT and a pulse interval of 5 ns. The computational performance evaluated at other pulse intervals is shown in S3 and S4 of the Supporting Information. We theoretically verified that the condition with *V*_G_ of 1.6 V gave the system the highest nonlinearity, as discussed in S5, Supporting Information.^[^ ^42^, ^49^ ^]^ To improve the ability to map in higher dimensional space, the number of nodes is increased by connecting the reservoir states under various *V*_G_ conditions. NMSE monotonically reduces as the number of nodes increases in magnetic fields of both 186 and 169 mT, as shown in Figure 3f. Then, the NMSE at 169 mT reaches the minimal value of 6.41 × 10^−5^. Figure 3g,h shows the comparative results for the training and test phases of the NARMA2 task with a magnetic field of 169 mT and a pulse interval of 5 ns. NMSE_var._ in the training and test phase are 1.92 × 10^−2^ and 2.25 × 10^−2^, respectively. NMSE_var._ variation at various *V*_G_ applications is shown in Figure 3i. Similar to the NMSE of the former task, the minimal NMSE_var._ is achieved at *V*_G_ = 1.6 V with a magnetic field of 169 mT and a pulse interval of 5 ns. As shown in Figure 3j, NMSE_var._ monotonically reduces as the number of nodes increases with magnetic fields of both 186 and 169 mT. Then, the NMSE_var._ at 169 mT reaches the minimal value of 9.53 × 10^−3^. It was confirmed that the system was not overfitting, as described in S6, Supporting Information. Note that these NMSE and NMSE_var._ were not averages obtained by multiple trials or by shuffling of the train and test data. Reliability and reproducibility were confirmed, as was the difference in computational performance resulting from data shuffling, all of which are described in S7 and S8, Supporting Information. In this verification, the lowest NMSE_var._ achieved in this study was further reduced to 7.30 × 10^−3^ for the NARMA2 prediction task.

**Figure 4:**
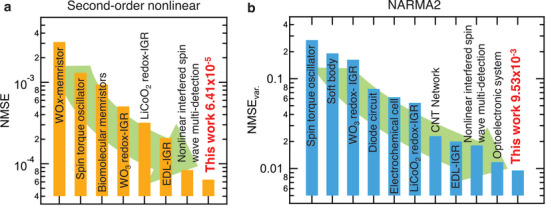
Benchmark comparison with other physical reservoirs. a) Comparison of NMSE of various devices. Also shown are the NMSEs of a WO*_x_*‐memristor,^[^ ^8^ ^]^ spin torque oscillator,^[^ ^16^ ^]^ biomolecular memristors,^[^ ^37^ ^]^ WO_3_ redox‐ion‐gating reservoir (IGR),^[^ ^34^ ^]^ LiCoO_2_ redox‐IGR,^[^ ^35^ ^]^ electric double layer (EDL)‐IGR,^[^ ^32^ ^]^ and nonlinear interfered spin wave multi‐detection.^[^ ^21^ ^]^ b) Comparison of NMSE_var._ of various devices. Also shown are the NMSEs_var._ of a spin torque oscillator,^[^ ^17^ ^]^ soft body,^[^ ^31^ ^]^ WO_3_ redox‐IGR,^[^ ^34^ ^]^ diode circuit,^[^ ^9^ ^]^ electrochemical cell,^[^ ^10^ ^]^ LiCoO_2_ redox‐IGR,^[^ ^35^ ^]^ carbon nanotube (CNT) network,^[^ ^11^ ^]^ EDL‐IGR,^[^ ^32^ ^]^ nonlinear interfered spin wave multi‐detection,^[^ ^21^ ^]^ and optoelectronic system.^[^ ^50^ ^]^

The NMSE and NMSE_var._ of various physical reservoirs are summarized in **Figure** **4**a,b, respectively. In both cases, the iono–magnonic reservoir showed the good performance, as follows: the NMSE of 6.41 × 10^−5^ and NMSE_var_ of 9.53 × 10^−3^ dramatically reduced by 76.3% on second‐order the nonlinear equation task and 52.7% on the NARMA2 task, compared to a previous study (NMSE of 8.37 × 10^−5^ and NMSE_var._ of 1.81 × 10^−2^).^[^ ^21^ ^]^ Then, the iono–magnonic reservoir updated the benchmark of both tasks. In particular, the iono–magnonic reservoir surpassed the excellent benchmark for an optoelectronic system (NMSE_var._ of 1.18 × 10^−2^)^[^ ^50^ ^]^ and established a new NARMA2 benchmark for the reported physical reservoirs.^[^ ^9^, ^10^, ^11^, ^17^, ^21^, ^31^, ^32^, ^34^, ^35^, ^50^ ^]^ Further, the NMSE_var._ of this study was comparable to the performance of deep reservoirs, which required complex hierarchical adjustments but improved computational performance (NMSE_var._ of 9.21 × 10^−3^).^[^ ^51^ ^]^ The improvements shown in this study are due to the excellent features of the iono–magnonic reservoir; that is, nonlinearity and the ability to map in higher dimensional feature space.

The Mackey–Glass prediction task, a benchmark task other than the NARMA task, was performed to verify whether the iono–magnonic reservoir could predict chaotic time series data.^[^ ^13^, ^25^, ^52^, ^53^, ^54^, ^55^, ^56^, ^57^, ^58^, ^59^ ^]^ The following equation represented the Mackey–Glass time‐delay derivative system:^[^ ^60^ ^]^

(4) $$\frac{dx\left(t\right)}{dt}=-\alpha x\left(t\right)+\frac{\beta x\left(t-\tau \right)}{x^{n}\left(t-\tau \right)+1}\;$$

Here, the respective constant variables are *α* = 0.1, *β* = 0.2, *n* = 10, and *τ* = 17.^[^ ^13^, ^25^, ^52^, ^53^, ^54^, ^55^, ^56^, ^57^, ^58^, ^59^ ^]^ As *τ* in the equation increased, the nonlinearity of the system increased. Then, the system behaved chaotically when *τ* ≥ 17. The fourth order Runge–Kutta method was used to solve this equation. The time step of the generated time series data was 5000, with the first 500 steps of each period discarded as the warming‐up period, the first 4000 steps as the training period, and the remaining 500 steps as the test period.

The generated time series data were converted into a pulsed signal, as in the NARMA task, and four different intervals were set to 5, 10, 15, and 20 ns. The magnetic fields applied when inputting the pulsed time series data were 169 and 186 mT. Eight different *V*_G_ conditions were set to 0, 0.2, 1.6, 2.0, 2.2, 2.4, 2.6, and 2.8 V, as in the NARMA task.

**Figure 5:**
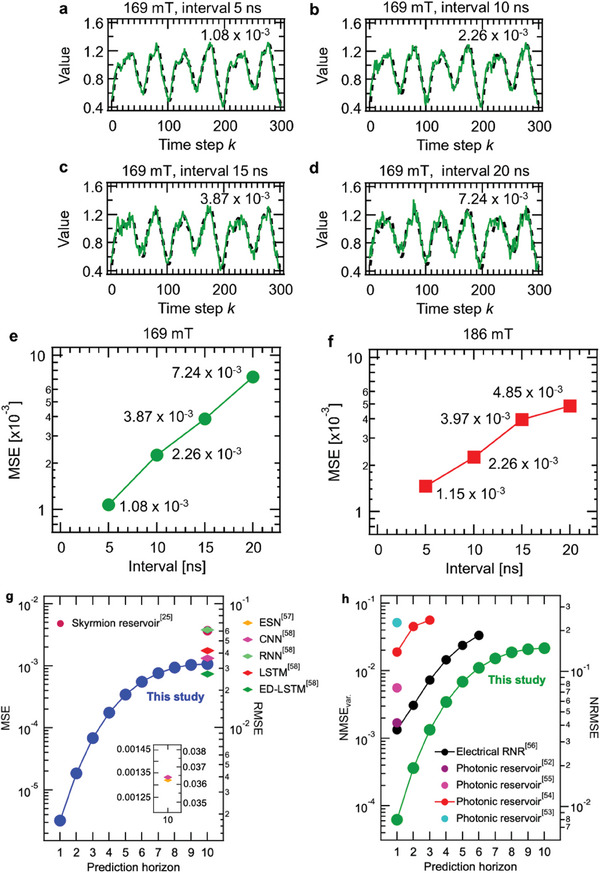
Prediction results for the Mackey–Glass time‐series data prediction task with magnetic fields and intervals of a) 169 mT and 5 ns, b) 169 mT and 10 ns, c) 169 mT and 15 ns, and d) 169 mT and 20 ns. The dashed black and green lines denote target and prediction waveforms, respectively. e,f) mean square errors (MSEs) variation at various intervals under magnetic fields of 169 mT (e) and 186 mT (f). g) Prediction horizon dependence of MSE and root mean square error (RMSE). h) Prediction horizon dependence at NMSE_var._ and normalized root mean square error (NRMSE). Also shown are the skyrmion reservoir,^[^ ^25^ ^]^ nanowire (NW) network reservoir,^[^ ^13^ ^]^ echo‐state network (ESN),^[^ ^57^ ^]^ convolutional neural network (CNN),^[^ ^58^ ^]^ recurrent neural network (RNN),^[^ ^58^ ^]^ long short‐term memory (LSTM),^[^ ^58^ ^]^ encoder‐decoder long short‐term memory (ED‐LSTM),^[^ ^58^ ^]^ electrical rotation neurons reservoir (RNR),^[^ ^56^ ^]^ and photonic reservoirs.^[^ ^52^, ^53^, ^54^, ^55^ ^]^

**Figure** **5**a–d show the target waveform and the predicted waveform ten steps ahead during the test period, at various pulse intervals. Under all conditions, the output waveform captured the characteristics of the target waveform well, with the output waveform being the smoothest at an interval of 5 ns. Figure 5e shows the interval dependence of a mean square error (MSE) for a ten‐step ahead prediction in a 169 mT magnetic field. As the interval increased, the MSE worsened. This trend is also shown in Figure 5f; when a magnetic field of 186 mT was applied, the MSE increased with increasing intervals, as was the case for the application of a 169 mT magnetic field. The lowest MSEs at a 5 ns interval and magnetic fields of 169 and 186 mT were 1.08 × 10^−3^ and 1.15 × 10^−3^, respectively. Figure 5g,h shows the prediction horizon dependence of the prediction error. The root mean square error (RMSE), NMSE_var._ and NRMSE, as well as the MSE, are shown in comparison to various physical reservoirs and simulated networks. The colored triangles represent the physical reservoirs, and the diamonds represent the simulated neural networks. Here, the networks are a long‐short term memory (LSTM) with a hidden layer consting of ten memory cells,^[^ ^58^ ^]^ an encoder–decoder LSTM (ED‐LSTM) with two LSTM networks and a time distributed layer,^[^ ^58^ ^]^ a convolutional neural network (CNN) with four hidden layers (i.e., a convolutional window size of 3, a max‐pooling window size of 2, a flattened layer, and a fully connected layer),^[^ ^58^ ^]^ a recurrent neural network (RNN) with two hidden layers including 20 neurons,^[^ ^58^ ^]^ and an echo‐state network (ESN) with a reservoir including 500 nodes.^[^ ^57^ ^]^ The iono–magnonic reservoir achieved lower prediction errors than physical reservoirs. On the ten step ahead prediction, MSE and RMSE of the iono–magnonic reservoir were 1.08 × 10^−3^ and 3.28 × 10^−2^, respectively. Other physical reservoirs used to predict the Mackey–Glass time‐series were the skyrmion (MSE of 3.70 × 10^−3^)^[^ ^25^ ^]^ and nanowire networks (NW) (RMSE of 9.40 × 10^−2^).^[^ ^13^ ^]^ RMSEs of the simulated representative neural network models used in this prediction were the LSTM (4.18 × 10^−2^)^[^ ^58^ ^]^ the CNN (3.64 × 10^−2^),^[^ ^58^ ^]^ the RNN (6.15 × 10^−2^)^[^ ^58^ ^]^ and the ESN (3.62 × 10^−2^).^[^ ^57^ ^]^ Further, the computational performance of the subject iono–magnonic reservoir was close to that of neural models modified for multi‐step ahead time series prediction (i.e., encoder–decoder LSTM) (RMSE of 2.71 × 10^−2^).^[^ ^58^ ^]^ On predictions other than ten steps ahead, the iono–magnonic reservoir was more precise than other physical reservoirs (i.e., the electrical rotating neurons reservoir (RNR) (NRMSEs of 1.37 × 10^−3^–3.42 × 10^−2^ for one to six steps ahead prediction)^[^ ^56^ ^]^ and photonic reservoirs (NMSEs_var._ of 1.70 × 10^−3^, 5.60 × 10^−3^, and 1.90 × 10^−2^ and NRMSE of 2.30 × 10^−1^ for 1 step ahead prediction)^[^ ^52^, ^53^, ^54^, ^55^ ^]^). Although there are theoretical neural network models (e.g., MultiTL‐KELM) with much higher performance dedicated to prediction,^[^ ^59^ ^]^ it was found that the iono–magnonic reservoir showed higher prediction performance than deep learning‐based networks and typical neural networks such as RNNs and ESNs. The number of nodes dependence of the errors of Mackey–Glass chaotic time‐series prediction task is described in S9, Supporting Information.

## 5 Improvements in the Ability to Map in a Higher Dimensional Space and Nonlinearity Achieved With an Iono–Magnonic Reservoir

**Figure 6:**
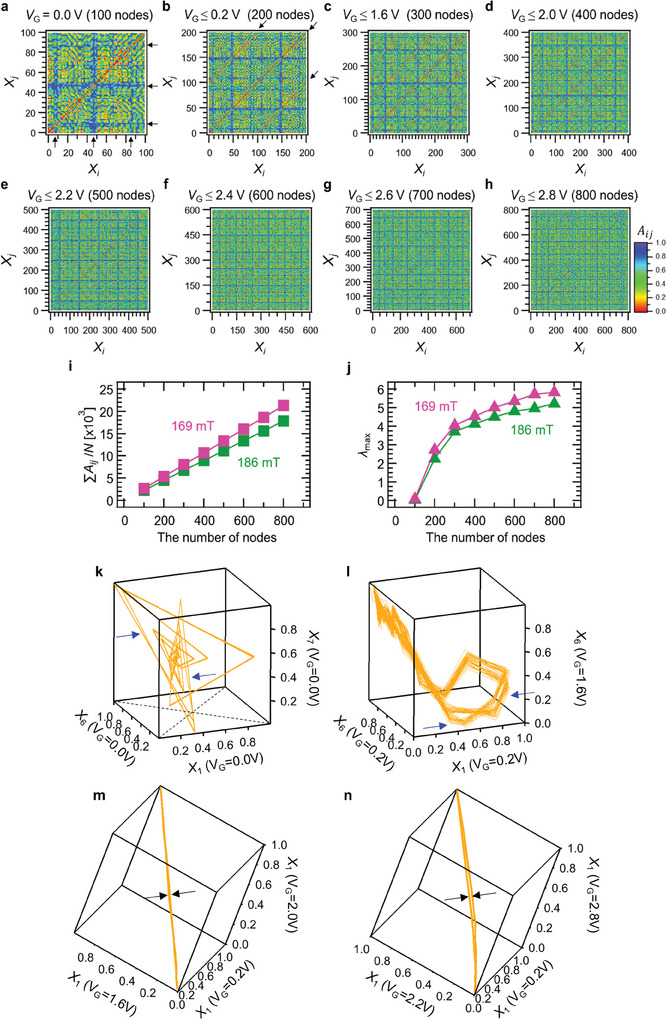
Evaluation of the ability to map in high dimensional space and the nonlinearity of the iono–magnonic reservoir. Non‐correlation coefficient (*A_ij_*) heatmaps of the iono–magnonic reservoir with a) *V*_G_ = 0.0 V (100 nodes), b) *V*_G_ ≤ 0.2 V (200 nodes), c) *V*_G_ ≤ 1.6 V (300 nodes), d) *V*_G_ ≤ 2.0 V (400 nodes), e) *V*_G_ ≤ 2.2 V (500 nodes), f) *V*_G_ ≤ 2.4 V (600 nodes), g) *V*_G_ ≤ 2.6 V (700 nodes), h) *V*_G_ ≤ 2.8 V (800 nodes), i) summation of *A_ij_* normalized by the number of nodes *N* as a function of the number of nodes, and j) maximum Lyapunov exponent *λ*_max_ as a function of the number of nodes. 3D trajectories k) at *X*_1_, *X*_6_, and *X*_7_ of *V*_G_ = 0.0 V; l) at *X*_1_ of *V*_G_ = 0.2 V, *X*_6_ of *V*_G_ = 0.2 V, and *X*_6_ of *V*_G_ = 1.6 V; m) at *X*_1_ of *V*_G_ = 0.2 V, *X*_1_ of *V*_G_ = 1.6 V, and *X*_1_ of *V*_G_ = 2.0 V; and n) at *X*_1_ of *V*_G_ = 0.2 V, *X*_1_ of *V*_G_ = 2.2 V, and *X*_1_ of *V*_G_ = 2.8 V. The solid blue arrows denote one of two trajectories described in the space, respectively. The solid black arrows denote the width of closed or open trajectories.

To verify the origin of the good performance, we evaluated the node state variation in the iono–magnonic reservoir.^[^ ^12^, ^34^, ^35^ ^]^ That is, the non‐correlation coefficients (*A_ij_*) between *X_i_* and *X_j_* at various numbers of nodes. *A_ij_* is defined as follows:^[^ ^12^, ^38^, ^39^ ^]^

(5) $$A_{ij}=1-\;r_{ij}=1-\frac{\mathrm{Cov}\left(X_{i},X_{j}\right)}{\mathrm{Var}\left(X_{i}\right)\mathrm{Var}\left(X_{j}\right)}$$

where *r_ij_* is the correlation constant, Cov(*X_i_*, *X*_j_) is the covariance between vectors *X_i_* and *X_j_*, and Var(*X*) ≅ Cov(*X*, *X*). Thus, there is a strong (weak) variation between *X_i_* and *X_j_* when *A_ij_* approaches 1 (0), indicating a high (low) ability to map in the higher dimensional space. **Figure** **6**a shows the non‐correlation coefficients (*A_ij_*) between *X_i_* and *X_j_* at *V*_G_ = 0.0 V (i.e., 100 nodes) in a magnetic field of 169 mT. There is a strong (weak) variation between *X_i_* and *X_j_* when *A_ij_* is close to 1 (0), indicating a high (low) ability to map in the higher dimensional feature space. The diagonal component (*A_ii_*) in Figure 6a is 0.0 because *i* = *j*. While most of the heatmap shows green and yellow, indicating that there are some correlations, there is a tendency for *A_ij_* to be high in ranges *X*_5_–*X*_10_, *X*_45_–*X*_50_, and *X*_85_–*X*_90_ and their vicinities, as shown by the solid black arrows in Figure 6a. These nodes do not correlate with others, but do strongly contribute to the ability of the reservoir to map in high‐dimensional space. The *A_ij_* at each *V*_G_ condition is shown in S10, Supporting Information.^[^ ^12^, ^34^, ^35^ ^]^

Figure 6b–h shows the *A_ij_* between *X_i_* and *X_j_* at various *V*_G_ combinations (200–800 nodes) in a magnetic field of 169 mT. At the diagonal component appearing every 100 nodes (i.e., *i* = *j*+100, *j*+200, *j*+300, *j*+400, *j*+500, *j*+600, and *j*+700), *X_i_* is similar to *X_j_*, as indicated by the solid black arrows in Figure 6b. This is due to the basic similarity of wave packets between each *V*_G_. However, this similarity is weakened when the number of combined *V*_G_ is increased, as shown in Figure 6d–h. This is due to large variations of wave packets in regions above a *V*_G_ of 2.0 V, as shown in Figure 2g–l. Figure 6i shows the normalized summation of *A_ij_*, which is an indicator of the ability of the reservoir to map in higher dimensional feature space when normalized to 100 nodes. Although the summation of *A_ij_* enhances as the number of combined reservoir states increases, in cases of both 169 mT and 186 mT, the slope of the summation of *A_ij_* in the case of 169 mT is particularly large in comparison to that of 186 mT, indicating that the ability of 169 mT to map in higher dimensional space is greatly developed in the scheme. This result is consistent with the result showing that the performance of the iono–magnonic reservoir was improved when the number of nodes was increased by spin wave manipulation. In addition to this, the maximum Lyapunov exponent *λ*_max_, which is an indicator of nonlinearity,^[^ ^21^, ^32^, ^61^, ^62^ ^]^ also increases as the number of combined reservoir states increases. Here, positive *λ*_max_ is defined as chaotic.^[^ ^63^, ^64^ ^]^ The *V*_G_s used to calculate *λ*_max_s in Figure 6j was 0.0, 0.2, 1.6, 2.0, 2.2, 2.4, 2.6, and 2.8 V. As shown in Figure 6j, these *V*_G_ applications illustrate the dependence of *λ*_max_ on the number of nodes used to improve high dimensionality. The number of nodes for each voltage condition is 100, which is added in order of decreasing voltage to generate the number of nodes from 100 to 800: 100 nodes have *V*_G_ = 0.0 V only; 200 nodes have *V*_G_ = 0.0 V and 0.2 V; 300 nodes have *V*_G_ = 0.0, 0.2, and 1.6 V. Finally, 800 nodes is the sum of nodes at all *V*_G_s. The origin of the chaotic nature of the iono–magnonic reservoir is the modulation of spin waves by the gate voltage, with nonlinear interference based on dipole interactions. As described in our previous work and reports on the spin‐wave interference reservoir using micromagnetic simulations, nonlinear interference due to magnetic dipole interaction is caused by the magnetic interaction of propagating spin waves with each other due to leakage fields, so a linear superposition of waves occurs, instead of a nonlinear superposition.^[^ ^21^ ^]^ By applying a large high‐frequency voltage to excite the spin waves, they are imparted a large amplitude, and their interaction with the surrounding spin waves becomes strong. Such strong dipole interaction is one of the causes of chaos. Modulation of the nonlinear interfered spin wave by a *V*_G_ changes the degree of magnetic dipole interaction. The interaction at *V*_G_ > 0 is relatively weaker than at *V*_G_ = 0.0 V because the application of a finite *V*_G_ reduces the saturation magnetization of YIG; at *V*_G_ = 0.0 V, the interfering spin wave is more chaotic (i.e., the positive maximum Lyapunov exponent *λ*_max_ is larger) due to the strong magnetic interaction, while the chaotic nature is also weaker (i.e., the positive λ_max_ is smaller) at a finite *V*_G_ because the magnetic interaction is weaker. This tendency can be seen from the *V*_G_ dependence of the *λ*_max_ shown in S10, Supporting Information, where the *λ*_max_ at *V*_G_ = 0.0 V under each magnetic field is more significant than those of other *V*_G_s. At *V*_G_ = 0.2 V and above, they are smaller than those at *V*_G_ = 0.0 V. Further, stronger nonlinear and more complex reservoir states can be generated by combining various chaotic reservoir states realized by *V*_G_ application. This corresponds to control of the chaotic nature of the system by *V*_G_ application.

The diversity of the reservoir state is large due to the combination of various dynamical systems generated under different *V*_G_ applied conditions. *λ*_max_ at a condition of 169 mT becomes larger than that at 186 mT in the entire region due to the multiple reservoirs generated by *V*_G_. It is found that multiple reservoirs successfully improve nonlinearity and the ability to map in high‐dimensional feature space. Figure 6k–n shows the trajectories in phase spaces created by selecting the axes as one of the cross sections of the 800‐dimensional phase spaces. Two trajectories with different diagonals in the *X*_1_–*X*_6_ plane can be seen on the subject space, and both trajectories have a finite width, as shown in Figure 6k. Such wider width trajectories are observed, as shown in Figure 6l. These are typical features of a chaotic nature.^[^ ^21^, ^32^, ^62^ ^]^ It can be seen that the degree of collapse of the collapsed trajectories in the two cross sections is different by comparison between Figure 6m,n, respectively. The trajectory composed of nodes, obtained with a voltage with a narrow range (e.g., 0.2, 1.6, and 2.0 V) is collapsed into a closed linear‐like shape. This closed trajectory results from a relatively weak chaotic state. On the other hand, the trajectory composed of nodes where a voltage with a wide range (e.g., 0.2, 2.2, and 2.8 V) is applied has an open shape compared to the former, indicating that such trajectory results from a relatively strong chaotic state. As this improvement of nonlinearity results from the diversity of the spin wave signal successfully manipulated over a wide *V*_G_ range, the iono–magnonic reservoir is given excellent ability to map in higher dimensional feature space and strong nonlinearity by utilizing such *V*_G_ range.

## 6 Evaluation of Complexity and Permutation Entropy of the Reservoir State of the Physical Reservoir

**Figure 7:**
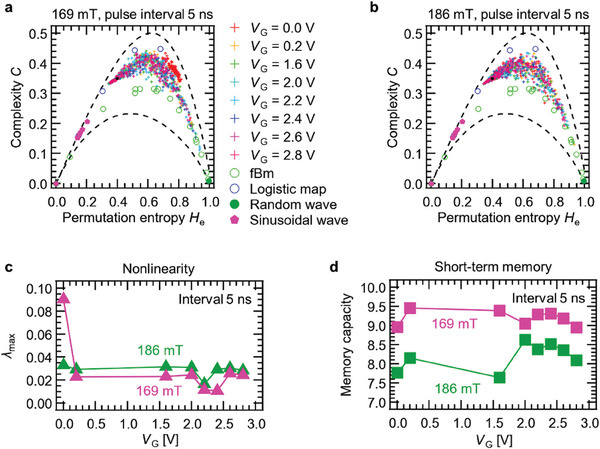
Evaluation of complexity *C* variation at various permutation entropies *H*_e_. The distribution of *C* and *H*_e_ with a) a 169 mT magnetic field and a pulse interval of 5 ns and b) a 186 mT magnetic field and a pulse interval of 5 ns. Also shown are plots of the fractional Brown motion (fBm), logistic map, random wave, and sinusoidal wave. The lower and upper dashed lines represent the lower and upper limits of *C*. c) Maximum Lyapunov exponent variation at various *V*_G_. d) Memory capacity variation at various *V*_G_.

Since chaos is not random but complex behaviors, complexity (*C*) and permutation entropy (*H*_e_) were introduced to evaluate whether the feature of time‐series data was complex or random.^[^ ^62^, ^65^, ^66^ ^]^ **Figure** **7**a,b shows *C* of the reservoir states of the iono–magnonic reservoir and various time‐series data as a function of *H*_e_. There is a minimum value and a maximum value of *C* (i.e., *C*_min_ and *C*_max_), and the distribution of the plot provides visual support for determining the characteristics of time‐series data (i.e., randomness, noisiness, periodicity, and chaos). Fractional Brownian motion (fBm) is chromatic noise, which is a stochastic process.^[^ ^65^ ^]^ On the other hand, *C* and *H*_e_ of the logistic map, which is known as a representative example of deterministic chaos by simple nonlinear mapping, distributes above the curve of chromatic noise.^[^ ^67^ ^]^ Thus, it can be ascertained whether the behavior of time‐series data is deterministic (chaotic) or stochastic when a plot locates above or below the fBm curve.^[^ ^67^ ^]^ If a signal shows periodic behavior, the combination of *H*_e_ and *C* is within the region of ≈0.1–0.2, as shown by the sinusoidal waves plotted in the figures. Based on the above relationship, we finally evaluated complexity *C* variation at various permutation entropy *H*_e_ of 800 nodes. When the interval was set at 5 ns, as shown in Figure 7a, a parabolic‐shaped distribution of the plots could be seen regardless of detector position and *V*_G_ amplitude, and the combinations of *C* and *H*_e_ of all nodes were plotted in the chaos region. Therefore, it can be said that the dynamics exhibit chaotic behavior, which is supported by the positive *λ*_max_ shown in Figure 6j. The plots of the reservoir state in a 186 mT magnetic field were parabola‐shaped distributions, as were those in a 169 mT magnetic field, as shown in Figure 6b, which also indicated chaotic behavior. Thus, it was concluded that the chaotic state, with strong nonlinearity, led to achieving the good computational performance in tasks where nonlinearity was emphasized, such as second‐order nonlinear equation task and the NARMA2 task. The detailed distribution of *C* is shown in S11, Supporting Information.^[^ ^7^, ^62^, ^65^, ^66^, ^68^ ^]^

The plots of 0.0 V at 169 mT in Figure 7a are clustered in a region of high complexity. The *λ*_max_ of 0.0 V at 169 mT was ≈0.09, which was the highest for all voltage conditions. For the other *V*_G_ conditions, *λ*_max_ was smaller than *V*_G_ of 0.0 V, and the complexity plot was parabolically distributed, unlike for a *V*_G_ of 0.0 V. This distribution was chaotic in nature: for permutation entropy *H*_e_ around 0.6, some nodes at *V*_G_ = 2.2 and 2.4 V had complexity close to that of stochastic fractional Brownian motion (fBm). The *λ*_max_ at *V*_G_ = 2.2 V and 2.4 V was small compared to the other *V*_G_ conditions. As shown in Figure 7b, the nodes at 186 mT also showed a parabolic complexity plot because each *V*_G_ condition had a *λ*_max_. With respect to the condition of 0.0 V at 186 mT which did not exhibit a small *λ*_max_ compared to the condition of 0.0 V at 169 mT, the complexity plot was found to be parabolically distributed, and the degree of nonlinearity inferred from the *λ*_max_ characterized the distribution of the complexity plot.

Figure 7c,d shows *λ*_max_ and memory capacity variations at various *V*_G_, respectively. The *V*_G_ dependence of the *λ*_max_ of 169 mT was smaller than that of 186 mT, except at *V*_G_ = 0.0 V of 169 mT. On the other hand, the short‐term memory capacity at 186 mT was smaller than that at 169 mT, as shown in Figure 7d. This is because the magnetic field of 169 mT (186 mT) had relatively smaller (larger) nonlinearity and larger (smaller) memory capacity. This result indicates a trade‐off between nonlinearity and memory capacity and that varying the magnetic field can tune the two features of the reservoir.

In the iono–magnonics, as the spin wave was measured in the steady state after the gate voltage was applied, the ion dynamics did not contribute to the memory capacity of the reservoir. Thus, the memory of reservoir computing using spin wave interference was caused by time decay of the spin wave. As described in theoretical studies and our previous work, it is known that the origin of short‐term memory in iono–magnonic reservoirs is the historic effect of spin wave reservoirs.^[^ ^19^, ^20^, ^21^ ^]^ The spin wave of the next time step arrived before the interfered wave of the past time step had decayed, thus developing a reservoir state that depended on past inputs and reservoir states. This historic effect contributed not only to the short‐term memory of the reservoir but also to its nonlinearity.^[^ ^19^, ^20^, ^45^ ^]^

The origin of the high performance of the iono–magnonic reservoir is a change in the spin wave properties of the YIG as a result of *V*_G_ application. Proton insertion induces electron doping and changes the magnetic properties, which contributes to manipulation of the amplitude and frequency of the spin wave. In the time multiplexing method for extracting nodes from the output waveform of a physical reservoir, manipulation of the waveform in the real‐time domain (e.g., the amplitude and frequency of the spin wave) changes the nonlinearity and short‐term memory of the nodes extracted from the nonlinear interfered spin wave; thus improving the higher dimension of the reservoir state. An iono–magnonic reservoir can generate reservoir states with various nonlinearities, short‐term memories, and complexities by manipulating *V*_G_. By combining the reservoir states generated by each *V*_G_ state, the *V*_G_ application effectively improves the high dimensionality of the reservoir, particularly when compared to conventional nonlinear interfered spin wave interference multi‐detection. *V*_G_ dependence and the number of nodes dependence of the computational performance are described in S12 and S13, Supporting Information.

## 7 Improvement of Operating Speed in the Iono–Magnonic Reservoir

In this study, waveforms were obtained by inputting 5000 steps of waveforms, with a pulse and an interval as one step, and integrating them 500 times. The pulse interval had five patterns of 2, 5, 10, 15, and 20 ns, and the ideal time required to acquire a single waveform of 5000 steps with 500 accumulations was 9–54 ms. Since measurements requiring this time are performed with eight *V*_G_ application states, the time required for eight waveform acquisitions was 72–432 ms. The waiting time was 1800 s from when the *V*_G_ was applied to the time of measurement, thus requiring 14 400 s to achieve the eight *V*_G_ states. The overall measurement time was (72–432 ms) + 14 400 s ≈ 14 400 s (i.e., 4 h). Nodes were extracted using the virtual node method,^[^ ^7^ ^]^ extracting 100 nodes per *V*_G_ state (i.e., 800 nodes/8 voltage states). However, actual time required, when using a multi‐channel oscilloscope to acquire a waveform with 5000 steps, was ≈61–87 s per voltage state. This time included 500 accumulations to average waveforms. Thus, the actual total time to extract 800 nodes was 14 888 s ( = 14 400 s + 488–696 s/8 voltage states). This time corresponded to times ranging from 4 h 8 min to 4 h 12 min. The actual measurement time required was much longer than in the ideal case described above, but the time to apply the voltage to change the magnetic properties of the YIG was still the rate‐limiting factor for an operating time.

Such operating time in the iono‐magnonic reservoir can be reduced by employing an array of eight devices. Eight combinations of two exciters, two detectors, and eight independent gate electrodes are fabricated on the YIG. For reservoir computing, using these eight *V*_G_ states, in which voltage is applied to eight independent electrodes in advance, the waiting time to apply *V*_G_ can be reduced, and the long computation times do not occur. This is because the eight combinations, consisting of a gate electrode and an antenna, operate independently and eliminate the need for waiting time when the *V*_G_ is changed. In addition, the eight reservoir states generated through each *V*_G_ state are acquired simultaneously, so it is necessary to measure the spin wave only once, instead of eight times. By this contrivance, eight *V*_G_ states are realized in advance, and the time used for the calculation is only the time taken (i.e., 61–87 s) for the spin wave to respond to the input signal.

An ideal operating time, accounting for integration time, falls within a relatively fast operating speed compared to other physical reservoirs, although the waveform integration increases the operation time. For example, the operating time of an optical circuit using high‐speed optical propagation as a reservoir is ≈2–78 ms, and a reservoir using spin waves (5000 steps and 500 integrations) has an operating time equivalent to this time (i.e., 9–54 ms).^[^ ^21^, ^27^, ^28^, ^29^, ^30^ ^]^ Further, the time required for 500 integrations of 5000 steps corresponds to less than 20 ms for one step in the ion‐gating reservoir.^[^ ^32^ ^]^ Therefore, even after 500 integrations, there is no significant degradation in operating speed compared to other physical reservoirs. The estimated operating times do not take into account the time associated with *V*_G_ application. This is because it is assumed that each *V*_G_ state has already been realized by applying the *V*_G_ in advance, using independent gate electrodes to realize multiple *V*_G_ states simultaneously. Here, the 500 integrations are done to improve the signal‐to‐noise ratio of the acquisition waveform. Using a material with a higher spin wave frequency or a larger magnetic moment may increase the spin wave signal and reduce this number of integrations. Electric power consumption, operating speed, robustness on the iono–magnonic reservoir, and the advantage of ion‐gating are described in S14 and S15, Supporting Information.^[^ ^8^, ^10^, ^11^, ^12^, ^21^, ^23^, ^27^, ^28^, ^29^, ^33^, ^34^, ^35^, ^36^, ^69^, ^70^, ^71^, ^72^, ^73^ ^]^

## 8 Conclusion

We demonstrated reservoir computing utilizing an iono–magnonic reservoir, which is an ion‐gating device that uses a solid‐state electrolyte and ferromagnetic material. For the first time, manipulation of propagating spin wave properties was achieved through modulation of the magnetic property using a solid‐state electrolyte. The iono‐magnonic reservoir exhibited excellent nonlinear transform abilities and the ability to map in higher dimensional space as a result of chaotic spin wave interference modulated by ion‐gating and achieved the good computational performance compared to other physical reservoirs thus far reported. There were no restrictions on the material or shape of the electrolyte or ferromagnetic materials that could be used in the iono–magnonic reservoir. In addition to reducing the electric power consumption and improving operating speed, miniaturization to an all‐thin‐film type would allow for a smaller device volume, which could in turn be integrated into electrical circuits, which would lead to high‐performance edge computing operation on terminal devices.

## 9 Experimental Section

### Fabrication of an Iono–Magnonic Device

A polished YIG single crystal with 111 orientation, which was grown by the floating zone technique, was supplied by MTI Co. (USA). The diameter and thickness of the single crystal were 5 and 0.5 mm, respectively. Coplanar waveguides, consisting of a 10 µm‐wide signal line and two 20 µm‐wide ground lines, were fabricated using conventional lithography. 10 nm thick‐Ti and 100 nm thick‐Au thin films were continuously deposited by electron beam evaporator. The distance between the edges of the antennas was 30 µm. The YIG and antenna dimensions were the same as those used in the previous work.^[^ ^21^ ^]^ Then, a Nafion with a Pt gate electrode thin film was attached to the YIG single crystal. *V*_G_ was defined as a voltage applied between the Pt gate electrode and ground with a potentio/galvanostat.

### Experimental Set‐Up to Detect Spin Waves

All experiments were performed in a high‐frequency signal measurement system, consisting of rf probes and an electromagnet, which was made by Toei Scientific Industrial Co., Ltd. An external magnetic field was applied perpendicular to the sample surface (i.e., the 111 direction of the YIG single crystal) to excite the FVMSW mode. The sample was kept at room temperature (i.e., 295 ± 1 K). The exciters and detectors shown in Figure 1a were connected, through rf probes, to an arbitrary waveform generator (Tektronix AWG5202) and a mixed signal digital oscilloscope (Tektronix MSO68B), respectively, to measure the excited spin waves at time domain. The exciters and detectors were arranged alternately on the YIG (i.e., Exciter A, Detector A, Exciter B, and Detector B). This configuration enabled spin waves excited at Exciter A and Exciter B to interfere with each other. Pulse voltage was input to the exciter to excite the spin waves. The amplitude of the pulse voltage was 400 mV. The input and output signals were amplified to 30 and 38 dB, respectively. To avoid detection of excess spin waves excited by previous sequences, long 4 µs intervals were inserted between each sequence. An average of 500 waveforms was taken to improve the signal‐to‐noise ratio (S/N). The repeating time would be reduced in future work, which was possible because the S/N could be improved by developing dramatically smaller signal‐amplifying electrical circuits. A vector network analyzer was connected to Exciter A and Detector A to conduct spin wave spectroscopy. Before the ion‐gating effect measurement, *V*_G_ was kept at each voltage used (0.0 V ≤ *V*_G_ ≤ 2.8 V) for 1800 s.

### Scanning Transmission Electron Microscopy (STEM) Combined With Electron Energy‐Loss (EEL) Spectroscopy

Two YIG single crystals, attached to Nafions, were prepared to acquire a cross‐sectional STEM image of the device and an EEL spectra. One was a YIG single crystal without a history of voltage application (‘Unbias’) and another was a YIG single crystal with a history of voltage application of 2.8 V between Pt electrode on Nafion and a bottom Au electrode deposited on the single crystal (“Bias”). After removing Nafions, the samples were coated by Pt deposition for protection in a sputtering system. The cross‐sectional lamella samples for STEM observation were prepared by a focused‐ion beam (FIB) after W coating. To avoid electron charging, the lamella samples were coated with 3 nm‐thick amorphous carbon. The STEM‐EEL spectra were obtained with a 200 kV scanning transmission electron microscope (JEOL, JEM‐2400FCS), equipped with a field‐emission gun and an EEL spectrometer (Gatan, GIF Continuum). The probe current was 97.6 pA. The energy dispersion and full width at half maximum of the zero‐loss peak were 0.15 eV pixel^−1^ and 0.9–1.2 eV, respectively. The entrance aperture of the spectrometer was 5 mm. The exposure time and integration time of the detector were ≈0.02 s pixel^−1^. The spectra were acquired at 2 nm steps.

### Nonlinear Time Series Data Prediction Task

In solving a second‐order nonlinear dynamical equation task, a random wave was input to a second‐order nonlinear dynamical system or NARMA2 system to obtain target waveforms for each task. The respective outputs *d*(*k*) and *d*(*k* + 1) from those dynamical and NARMA2 systems at discrete time *k* are described as follows:^[^ ^5^, ^8^, ^9^, ^10^, ^11^, ^12^, ^16^, ^17^, ^21^, ^22^, ^31^, ^32^, ^33^, ^34^, ^35^, ^37^, ^48^ ^]^

(6) $$d\;\;\;\;\left(k\right)=0.4d\;\;\left(k-1\right)+0.4d\;\;\left(k-1\right)d\;\;\left(k-2\right)+0.6u^{3}\;\;\left(k\right)+0.1\;$$

and

(7) $$d\;\;\;\;\left(k+1\right)=0.4d\;\;\left(k\right)+0.4d\;\;\left(k\right)d\;\;\left(k-1\right)+0.6u^{3}\;\;\left(k\right)+0.1$$

The outputs from systems *d*(*k*) and *d*(*k* +1) depended not only on the current input *u*(*k*) but also on the past states by two steps, which were *d*(*k−*1) and *d*(*k−*2) for Equation (6) and *d*(*k*) and *d*(*k*−1) for Equation (7). The second terms on the right‐hand sides of these equations were the cross terms that made them second‐order nonlinear systems. Thus, the reservoir was required to have nonlinearity and short‐term memory in order to solve these tasks. Here, the *u*(*k*) ranged from 0.0 to 0.5.

The subject reservoir computing system was trained and tested with a random waveform to predict the output from a second‐order nonlinear dynamic system and a NARMA2 model. Before being input to the reservoir system, the *u*(*k*) was transformed into a pulsed signal with various intervals of 2–20 ns. Each of these signals was input to Exciter A or Exciter B of the iono–magnonic reservoir to which perpendicular magnetic fields of 169 and 186 mT were being applied. These magnetic fields were the conditions that gave the lowest NMSE and NMSE_var._ in the previous study. The voltages induced by a spin wave, which reached each detector, were measured by an oscilloscope, and 50 nodes per detector were from each induced voltage. Thus, 50 reservoir states (w/o multi‐detection) or 100 reservoir states (with multi‐detection) were obtained by the reservoir from 1D input *u*(*k*). Time steps were separated into training phases with a time step of 3500 and test phases with a time step of 500, after the first time step of 1000 was discarded. In the training phase, the weight parameters combining the reservoir state and the readout node were optimized to minimize the difference between the target waveform output *d*(*k*) from the theoretical model (i.e., Equations (6) and (7)) and the reservoir output *y*(*k*). Thus, the weight coefficient *W_i_* in *y*(*k*) was optimized to correspond to *d*(*k*). This is described as follows:

(8) $$y\;\;\;\;\left(k\right)=\sum_{i=1}^{n}W_{i}X_{i}\left(k\right)+b$$

Here, *n* and *b* were the total number of nodes and a bias term, respectively. *w*_i_ was optimized by ridge regression as training for the system.

### Ridge Regression for Solving Second‐Order Nonlinear Dynamic Equation Tasks and NARMA2 Tasks

In the time series data equation tasks, the readout network of the nonlinear interfered spin wave multi‐detection reservoir was trained by ridge regression. The reservoir output *y*(*k*) shown in Equation (8) was transformed to:

(9) $$y\;\;\;\;\left(k\right)=W•X\left(k\right)$$

Here, ***W *** = (*w*_0_, *w*_1_, …, *w_n_*) and ***X*** (*k*) = (*X*_0_(*k*), *X*_1_(*k*), …, *X*_n_(*k*))^T^ are the weight vector and the reservoir state vector with a reservoir size of *n*, respectively. Note that *w*_0_ = *b* and *X*_0_(*k*) = 1 to introduce the bias *b* shown in Equation (8). The cost function *J*(***W***) in the ridge regression was defined as follows:

(10) $$J\;\;\;\;\left(W\right)=\frac{1}{2}\;\sum_{k=1}^{T}{\left(y_{t}\left(k\right)-y\left(k\right)\right)}^{2}+\frac{\beta }{2}\sum_{i=0}^{N}{w_{i}}^{2}$$

where *T*, *𝛽*, and *y*_t_(*k*) are the data length in the training phase, the ridge parameter, and the target waveform generated by Equations (6) or (7), respectively. *T* = 3500 and *𝛽* = 0 were fixed for all the tasks. The weight matrix $\hat{W}$, which minimizes cost function *J*(***W***), was given by the following equation;

(11) $$\hat{W}=YX^{T}{\left(XX^{T}+\lambda I\right)}^{-1}\;$$

Here, ***Y *** = (*y*_t_(1), *y*_t_(2), …, *y*_t_(*T*)); ***X*** (*k*) = (*X*(1), *X*(2), …, *X*(*T*)); and ***I*** (⊆ ℝ^(^ *^N^* ^+1)×(^ *^N^* ^+1)^) are the target output vector, the reservoir state matrix, and the identify matrix, respectively.

Then, after learning the readout weight, the computational performance was evaluated by “NMSE” to compare the performance of the reservoir system with that of other systems. The NMSE for the second‐order nonlinear dynamical equation task was described as follows;

(12) $$\mathrm{NMSE}\;=\frac{\sum_{k=1}^{T}{\left(d\left(k\right)-y_{p}\left(k\right)\right)}^{2}}{\sum_{k=1}^{T}{\left(d\left(k\right)\right)}^{2}}\;$$

Here, *T*, *d*(*k*), and *y*_p_(*k*) are the lengths in the training phase (*T *= 3500) or test phase (*T* = 500), the target signal, and the predicted signal, respectively. The NMSE_var._ for the NARMA2 task was described as follows;

(13) $$\mathrm{NMS}E_{\mathrm{var}.}=\frac{\sum_{k=1}^{T}{\left(d\left(k\right)-y_{p}\left(k\right)\right)}^{2}}{\sum_{k=1}^{T}{\left(d\left(k\right)-d_{\mathrm{ave}.}\right)}^{2}}\;$$

where *d*_ave._ is the time average of *d*(*k*).

### Prediction Errors for the Mackey–Glass Chaotic Time‐Seires

The prediction errors for the Mackey–Glass chaotic time‐series were described as follows;

(14) $$\mathrm{MSE}\;=\frac{\sum_{k=1}^{T}{\left(x\left(k\right)-y_{p}\left(k\right)\right)}^{2}}{T}\;$$

(15) $$\mathrm{RMSE}\;=\sqrt{\frac{\sum_{k=1}^{T}{\left(x\left(k\right)-y_{p}\left(k\right)\right)}^{2}}{T}}\;$$

and

(16) $$\mathrm{NRMSE}\;=\sqrt{\frac{\sum_{k=1}^{T}{\left(d\left(k\right)-y_{p}\left(k\right)\right)}^{2}}{\sum_{k=1}^{T}{\left(d\left(k\right)-d_{\mathrm{ave}.}\right)}^{2}}}\;$$

### Micromagnetic Simulation

To investigate nonlinearity variation at various pulse intervals, a theoretical simulation was performed using a Mumax3 micromagnetic simulator.^[^ ^49^ ^]^ YIG with 380 µm × 90 µm × 0.12 µm was used for the spin wave waveguide to investigate the spin dynamics near a surface region in the vicinity of an antenna. The two exciters used consisted of a signal line (10 µm × 90 µm × 0.12 µm) and two ground lines (20 µm × 90 µm × 0.12 µm). The detection areas of the two detectors were 10 µm × 90 µm × 0.12 µm, which corresponded to the signal lines of the detectors. The mesh was cubic, measuring 40 nm × 40 nm × 40 nm along Cartesian coordinates defined by the origin at the center of the surface plane on the YIG. A spin with a saturation magnetization of 157.9 kA m^−1^ was located at every mesh corner. The simulation time step was 10 ps. The material parameters estimated from experimental measurement at *V*_G_ = 0.0, 0.2, 1.6, and 2.8 V were a saturation magnetization of 157.9, 153.5, 148.3, and 148.5 kA m^−1^ and a uniaxial anisotropy along the *z*‐axis *K*_U_ of −6.32, −5.94, −5.73, and −5.75 kJ m^−3^. To simulate spin wave property variation at various *V*_G_, these parameters were set to various values corresponding to the experimental result shown in Figure 1e. A cubic magneto‐crystalline anisotropy of 0.0, an exchange stiffness constant of 3.7 pJ m^−1^, and a damping constant of 1 × 10^−4^ were used as the typical values of YIG. A static magnetic field of 0.3 T was applied along the *z*‐axis (i.e., perpendicular to the YIG surface) over the entire region. An excitation field, with a rectangular shape‐pulse, was set to 80 mT along the *y*‐axis of the exciters applied in the exciter. The field was estimated as a simulation result shown in the literature.^[^ ^42^ ^]^ The pulse interval was set at 5 ns, which was the best condition for time‐series data prediction tasks. The field vectors at the signal and ground lines were positive and negative, respectively, because electric current flowed in the opposite direction.

### Lyapunov Spectrum

The Lyapunov spectrum is an index used to evaluate orbital instability, which is one of the features of chaos. Lyapunov exponents of nonlinear interfered spin wave multi‐detection were calculated by the Jacobi matrix method.^[^ ^62^ ^]^ The Lyapunov exponent *λ* was defined as follows:^[^ ^62^ ^]^

(17)

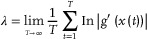




Here, *t* and *g*′(*) were iteration time and differentiation of mapping function *g*(*), respectively. If *λ* was positive (negative), proximity orbits were detached (asymptotic). In calculation, the Lyapunov exponent was calculated by taking a hypersphere (*ε*‐sphere) in m‐dimensional space of minute radius *ε* around a point, assuming that its change after one step could be approximated linearly, and then estimating the Jacobi matrix using that variation.

The displacement vector ***µ***_i_ ∈ R^N+L^ with respect to ***v*** (*k*_i_) as seen from ***v*** (*t*), was expressed as;

(18) $$\mu_{i}=v\left(k_{i}\right)-v\left(t\right)$$

Since *v*(*t*) and *v*(*k*_i_) transit *v*(*t* + s) and *v*(*k*_i_ + s) when time *s* elapses, the displacement vector ***z***_i_ at time *t* + *s* could be described as:

(19) $$z_{i}=v\left(k_{i}+s\right)-v\left(t+s\right)$$

Under the consumption that *ε* and *s* are small enough so as to be negligible, ***µ***_i_ and ***z***_i_ could be linearly approximated as follows:

(20) $$z_{i}=\hat{J}\;\left(t\right)\mu_{i}\;$$

where $\hat{J}\left(t\right)$ is the Jacobi matrix to be estimated. Thus, the Jacobi matrix could be estimated as follows;

(21) $$\hat{J}\;\;\;\;\left(t\right)=z_{i}\;\mu_{i}^{T}{\left(\mu_{i}\mu_{i}^{T}\right)}^{-1}\;$$

$\hat{J}\left(0\right)$ that is $\hat{J}\left(t\right)$ at *t* = 0 is described by QR decomposition, as follows;

(22) $$\hat{J}\;\;\;\;\left(0\right)=Q_{1}\;R_{1}\;$$

where ***Q*** and ***R*** are the orthogonal matrix and the upper triangular matrix. At time *t* + 1, multiply $\hat{J}\left(t\right)$ by the orthogonal matrix from one time earlier to obtain the following relation;

(23) $$\hat{J}\;\;\;\left(t\right)\;Q_{t}=Q_{t+1}\;R_{t+1}$$

Using the upper triangular matrix at each time obtained in this way, the *λ* obtained was as follows;

(24) $$\lambda_{i}=\lim_{T\to \infty }\frac{1}{2T}\;\sum_{t=1}^{T}\ln\left|R_{k}^{ii}\right|$$

Here, $R_{k}^{ii}$ is the *i*‐th diagonal element of the upper triangular matrix *R_k_*.

### Short‐Term Memory

A random wave utilized on NARMA tasks was used for this task and input to the iono–magnonic reservoir. The first half of the 500 time steps was discarded. The target output *d*(*k*) was *u*(*k*‐*τ*), which was a time series data delayed time step of *τ*. The ***W***_out_ in the reservoir network was optimized using the training set. The system predicted on the test set, and the square of the correlation coefficient *r* ^2^(*k*) between the ideal targets *d*(*k*) and the model predictions *y*(*k*) was determined as follow;

(25) $$r^{2}\left(k\right)=\frac{Cov{\left(d\left(k\right),y\left(k,\tau \right)\right)}^{2}}{Var\left(d\left(k\right)\right)\times Var\left(y\left(k,\tau \right)\right)}$$

Here, Cov(A, B) is the covariance between vectors **A** and **B**, and Var(A) ≅ Cov(A, A). *r* ^2^ is [0,1]. *r* ^2^ = 1 means that the reservoir reconstructed the *d*(*k*) completely. The short‐term memory capacity (MC) was then calculated by taking the sum of *r* ^2^(*k*) over the range of delays. In this study, the maximum step delay for a memory capacity evaluation was set to 20. This step delay was used to avoid integrating meaningless backgrounds. MC is described as follow,

(26) $$\mathrm{MC}\;=\sum_{\tau =1}^{20}r^{2}\;\;\;\;\left(\tau \right)$$

## Conflict of Interest

The authors declare no conflict of interest.

## Supporting information



Supporting Information

## Data Availability

The data that support the findings of this study are available from the corresponding author upon reasonable request.
