# Supplementary material for: Iono–Magnonic Reservoir Computing With Chaotic Spin Wave Interference Manipulated by Ion‐Gating
Source: Adv Sci (Weinh). 2024 Nov 17;12(3):2411777. doi: 10.1002/advs.202411777 (PMC11744637; doi:10.1002/advs.202411777)
Supplement: Supplementary file 1 — Supporting Information [file ADVS-12-2411777-s001.docx]

Supporting Information

**Iono-magnonic Reservoir Computing with Chaotic Spin Wave Interference Manipulated by Ion-Gating**

*Wataru Namiki^1^, Daiki Nishioka^1,2^, Yuki Nomura^3^, Takashi Tsuchiya^1*^, Kazuo Yamamoto^3^, and Kazuya Terabe^1^*

^1^Research Center for Materials Nanoarchitectonics (MANA), National Institute for Materials Science, 305-0044, Ibaraki, Japan

^2^Faculty of Science, Tokyo University of Science, 125-8585, Tokyo, Japan

^3^Nanostructures Research Laboratory, Japan Fine Ceramics Center, 2-4-1 Mutsuno, Atsuta, Nagoya, Aichi, 456-8587, Japan

e-mail: TSUCHIYA.Takashi@nims.go.jp

**S1. Magnetic property of the iono-magnonic device**

**Figure S1**a is a schematic illustration of an iono-magnonic device with a Y_3_Fe_5_O_12_ (YIG) single crystal and Nafion and a configuration that is used to measure spin wave property variation at various *V*_G_. Port 1 and port 2 of a vector network analyzer (VNA) are connected to Exciter A and Detector A, respectively. An input signal of 5dBm was swept from 1 MHz to 8 GHz. A magnetic field runs along the surface plane in a direction perpendicular to the YIG surface (i.e., ***H*** // 111). Frequency *f* of a forward volume magnetostatic wave is described as follow:

$$f=\gamma\sqrt{\left( H-H_{a} \right)\left\{ \left( H-H_{a} \right)+M_{S}\left( 1-\frac{1-e^{-kd}}{kd} \right) \right\}}. \left( S1 \right)$$

Here, *γ*, *H*, *H*_a_, *M*_S_, *k*, and *d* are gyromagnetic ratio, applied external field, magnetic anisotropy field, saturation magnetization, wave number of spin wave, and YIG thickness, respectively. *γ* was set to 28 GHz/mT.^[S1,S2]^ Magnetization (*M*) – magnetic field (*H*) curve of a pristine YIG single crystal is shown in Figure S1b. ***H*** aligns with the surface plane of the YIG. The measured saturation magnetization (*M*_S_) was 198.4 mT. This value was referred to for the fitting of the magnetic field dependence of the spin wave frequency. The *V*_G_ dependence of *M*_S_ and *H*_a_ are obtained from the fitting using **equation S1**. The fitting results for the magnetic field dependence of spin wave frequency are shown in Figures S1b-i. These are in good agreement with the experimental results. Here, the frequency variation induced by *V*_G_ application looks very small when the plots are displayed in the GHz range, since the maximum frequency variation induced by *V*_G_ application is approximately 20 MHz. However, the difference in spin wave frequency is clearly shown in Figures 2 a-f.


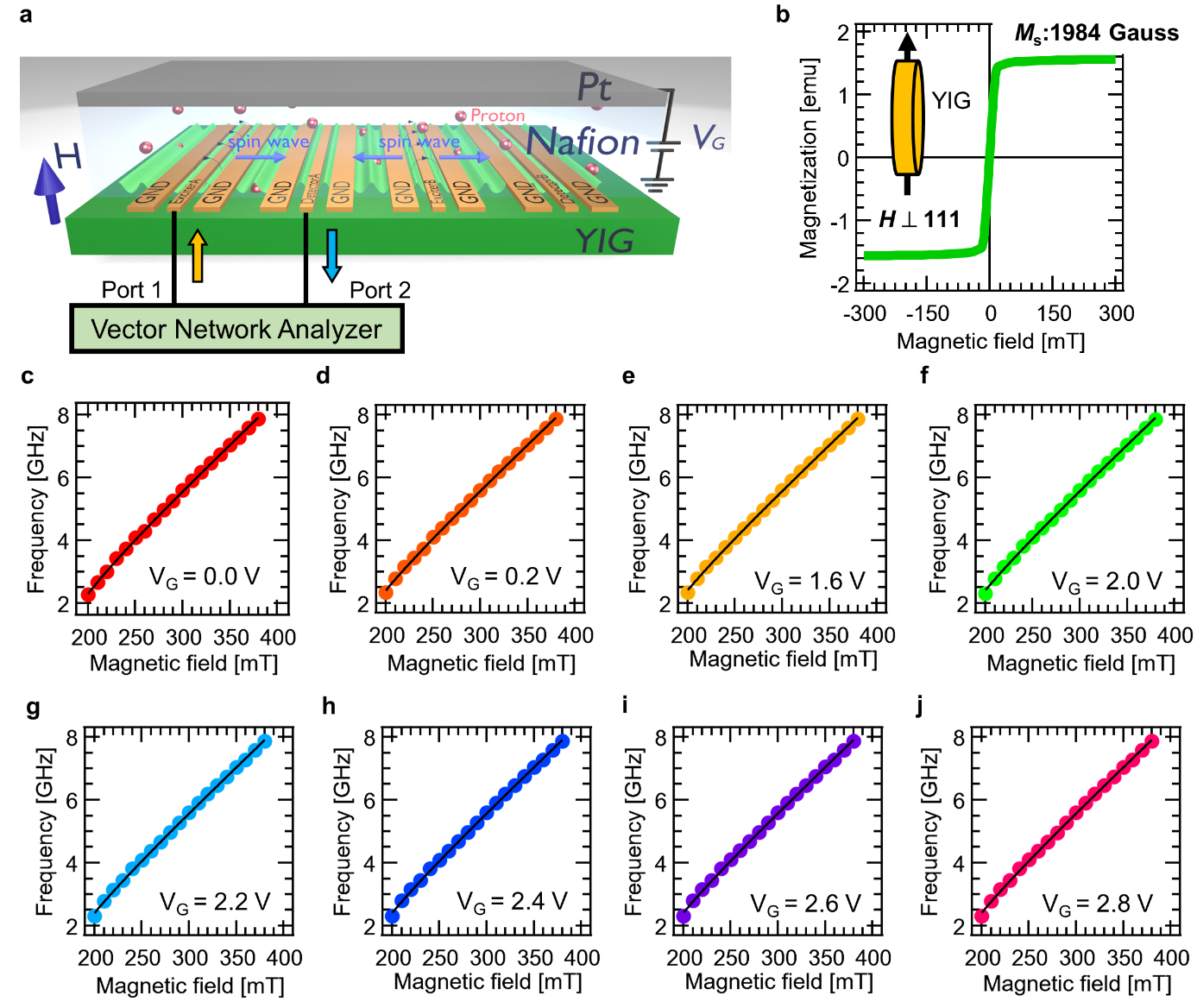


**Figure S1.** A schematic illustration of an iono-magnonic device, and its measurement configuration, for spin wave property manipulation by ion-gating. a) n iono-magnonic device with Y_3_Fe_5_O_12_ (YIG) single crystal and Nafion and its experimental configuration with a vector network analyzer. b) Magnetization as a function of applied in-plane magnetic field *H* for YIG single crystal (*H* ⊥ 111). Magnetic field dependence of spin wave frequency at c) *V*_G_ = 0.0 V, d) *V*_G_ = 0.2 V, e) *V*_G_ = 1.6 V, f) *V*_G_ = 2.0 V, g) *V*_G_ = 2.2 V, h) *V*_G_ = 2.4 V, i) *V*_G_ = 2.6 V, j) *V*_G_ = 2.8 V. The solid black line denotes the fitting result using equation S1.

Here, the spin wave signal is buried in a background spectrum based on the ambient environment and a measurement circuit (connectors, cables, and probes). **Figure S2** shows the spin wave spectra acquired at *H* = 0 and 170 mT and a de-embedded spectrum. A target spin-wave signal (*S*_21_SW_) is embedded (or buried) in an acquired spectrum (*S*_21_) containing a background spectrum (*S*_21_BG_). By subtracting from the *S*_21_ spectrum the background spectrum (*S*_21_BG_), acquired at a zero magnetic field and corresponding to *S*_21_(*H* = 0), a spectrum containing only the spin wave signal (*S*_21_SW_) can be extracted (i.e., de-embedded). De-embedded *S*_21_, under application of magnetic field *H*, is described as follows;

De-embedded *S*_21_(*H*) = S_21_SW_(*H*) = *S*_21_(*H*) – *S*_21_(*H* = 0). (S2)


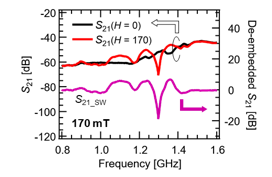


**Figure S2**. Spin wave spectra acquired at H = 0 and 170 mT, and its de-embedded spectrum.

**S2. Manipulated nonlinearity of spin wave, and high dimensionality**

**Figures S3**a and b show nonlinear spin wave interference at *V*_G_ = 0.0 V. A spin wave signal is varied at Detector A and Detector B, even though the same exciter (Exciter A) is used to excite the spin wave, as shown in Figure S3a. As shown in Figure S3b, there is a finite difference between the interfered spin wave signal and a linear summation of these spin wave signals. This difference shows that the interfered spin wave has nonlinearity. The intercepted spin wave variation detected at Detector A at various *V*_G_s is shown in Figure S3c. Large variations of frequency and/or phase are observed over the entire time domain, as shown in Figures S3d and e. Similar variations are shown in the spin wave signal detected at Detector B, as shown in Figures S3f-h. This variation results from the spin wave property modulated by the *V*_G_ application. Difference variations under various magnetic fields are summarized in Figure S3i. Nonlinear component amplitude is decreased as *V*_G_ increases, as shown in Figure S3j. Nonlinear spin wave interference originates as dipole-dipole interactions between spin precessions. Since the spin magnetic moment in an Fe ion is electrochemically reduced by a doped electron, the interaction may become weaker. This change contributes to the degree of nonlinearity of the interfered spin wave. The spin wave manipulation achieved with the iono-magnonic device contributes to the improvement of nonlinearity and the ability to map in higher dimensional space as a reservoir, as shown in Figure S3k.

In the iono-magnonic reservoir, the magnetic properties are changed by proton insertion and electron doping into YIG. This scheme can manipulate from the interface at the ferromagnetic material/solid electrolyte to a relatively large region. This can increase the controllable volume magnetization and thus the detectable spin wave intensity. For example, voltage-controlled magnetic anisotropy (VCMA) is a promising method for fast magnetization manipulation, with low electric power consumption.^[S3,S4]^ However, since the magnetic properties are controlled via a shift in the Fermi level at the surface of a ferromagnetic material, the ferromagnetic material must be extremely thin. This ferromagnetic volume reduction weakens the intensity of the detected signal, which may degrade the computational performance due to the reduction of the signal-to-noise ratio.


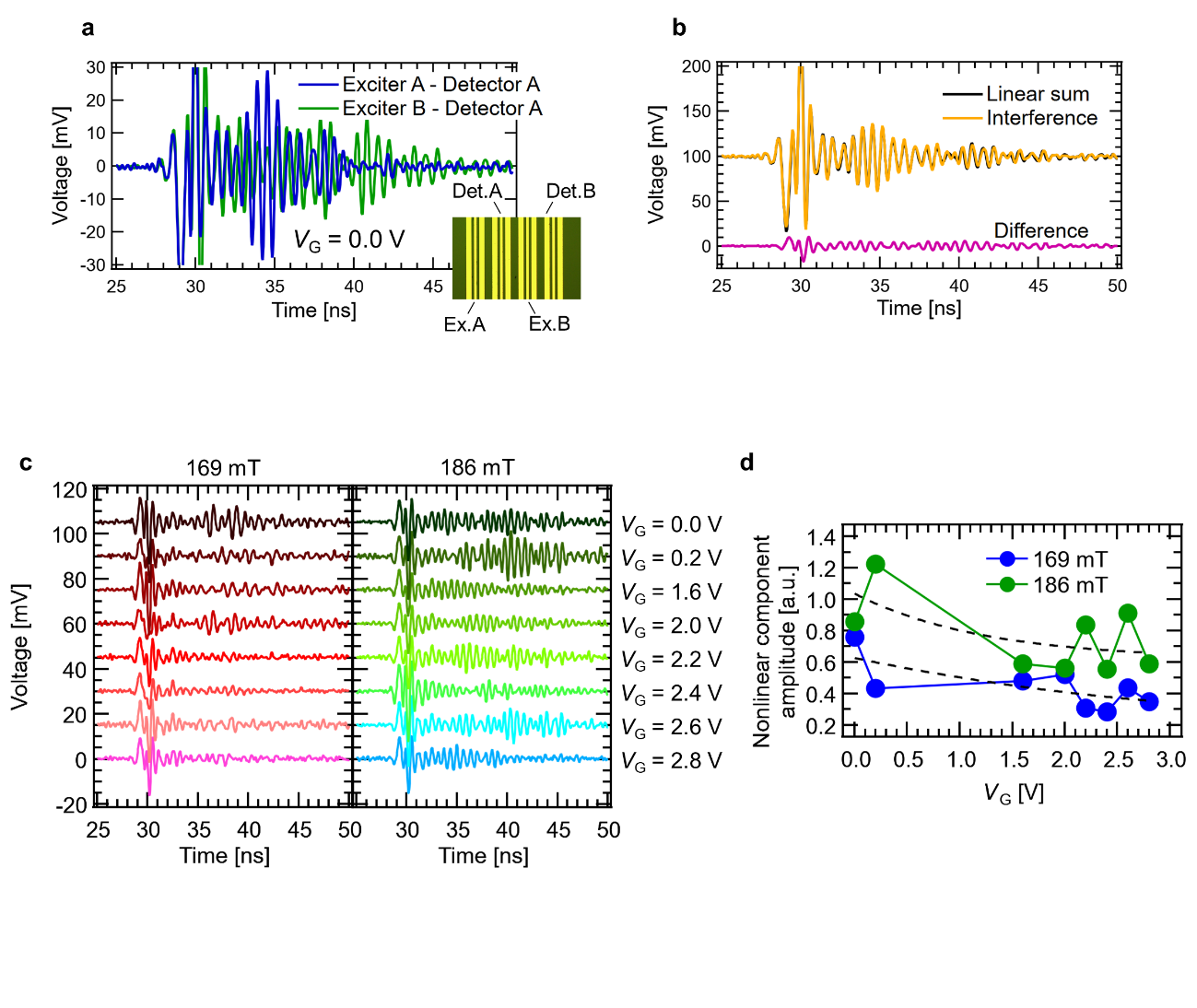


**Figure S3.** Nonlinearity of an interfered spin wave. a) Voltage induced by spin waves propagating between Exciter A (Ex. A) and Detector A (Det. A) and between Exciter B (Ex. B) and Detector A (Det. A). b) Compared voltage induced by an interfered spin wave and a linear combination of the two spin waves shown in (a). c) The waveform of the nonlinear components at various *V*_G_ at 169 mT (left-hand panel) and 186 mT (right-hand panel). d) Nonlinear component variation at various *V*_G_ at 169 mT and 186 mT. The dashed lines denote guidelines.

**S3. Error variations of the second-order nonlinear dynamic equation task and the NARMA2 task, at various pulse intervals**

**Figures S4**a and b show the NMSE and NMSE_var._ of a second-order nonlinear dynamic equation task and a second-order nonlinear autoregression moving average (NARMA2) task, respectively, in a magnetic field of 169 mT. Except for the 2 ns pulse interval, there is a tendency for both NMSE and NMSE_var._ to decrease as the pulse interval is shortened. Then, NMSE and NMSE_var._ reach the minimum values of 1.26 × 10^-4^ and 2.25×10^-2^ at an interval of 5 ns. As shown in Figures S4c and d, a similar tendency can be observed in normalized mean square error (NMSE) and NMSE using variance (NMSE_var._) of the tasks with a magnetic field of 186 mT, although the errors at the 2 ns pulse interval are close to those at the 5 ns interval. The minimum values of NMSE and NMSE_var._ under this condition are 1.40 × 10^-4^ and 2.70 × 10^-2^, respectively. Thus, the best condition for those tasks is a pulse interval of 5ns. Figures S4e and f show the number of nodes dependence of NMSE and NMSE_var._ of the second-order nonlinear equation task and the NARMA2 prediction task on the optimal conditions. The errors successfully reduced as the number of nodes increased, until the errors reached their lowest values (i.e, NMSE of 1.26 × 10^-4^ and NMSE_var._ of 2.25 × 10^-2^).


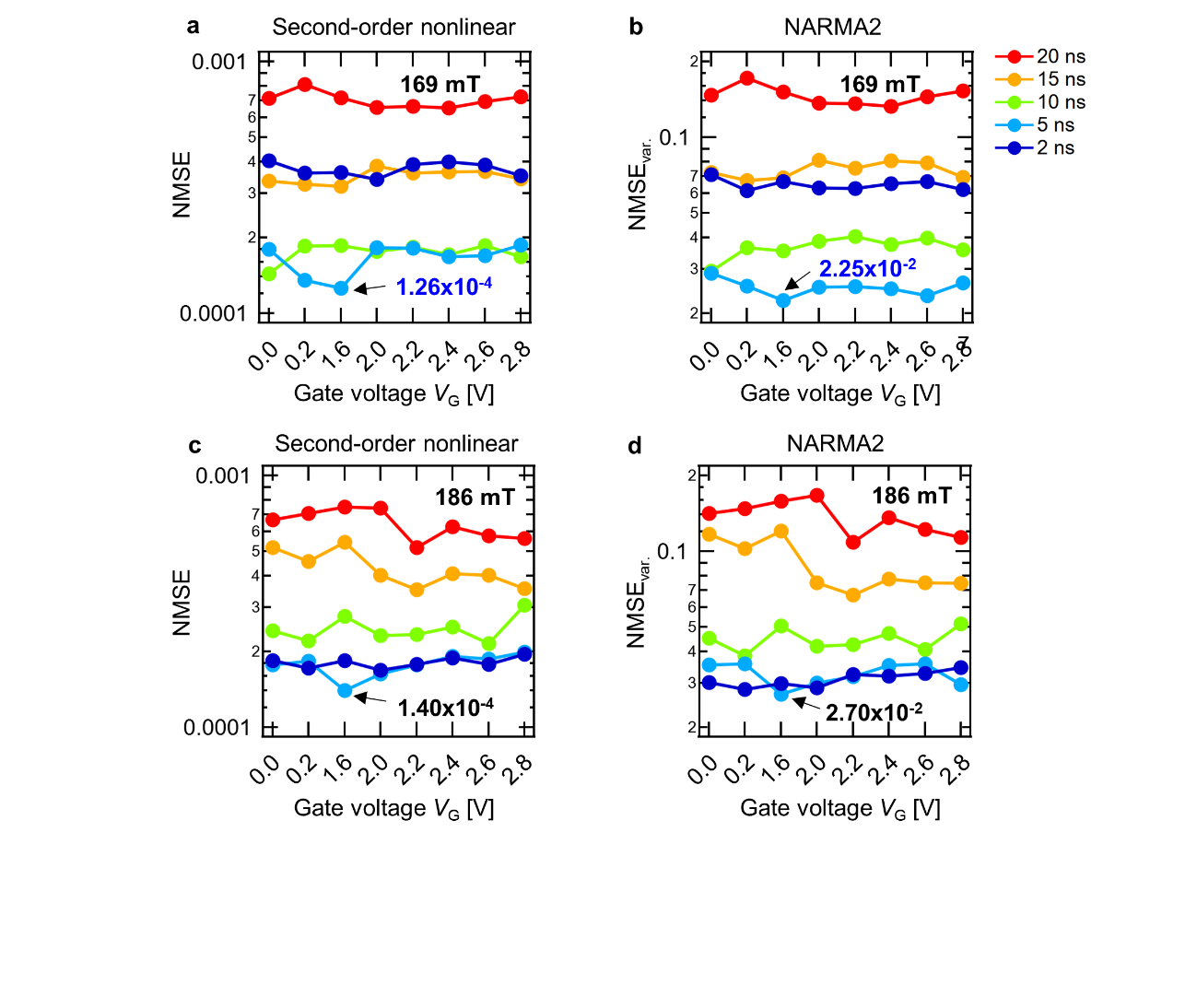

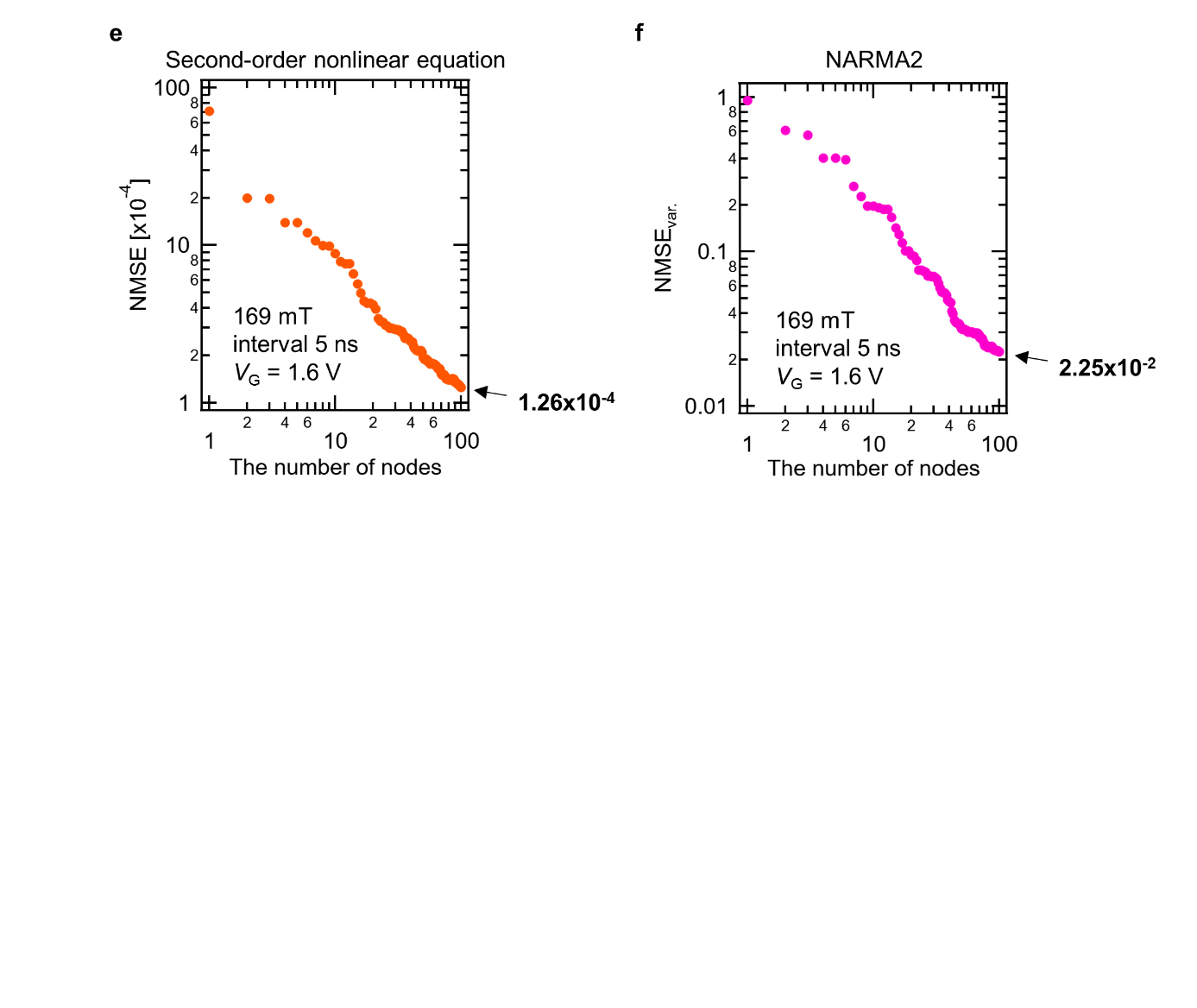


**Figure S4.** The result of time-series processing tasks. NMSE and NMSE_var._ variations at various gate voltage *V*_G_ of a) a second-order nonlinear equation task and b) a NARMA2 task, respectively, in a magnetic field of 169 mT. NMSE and NMSE_var._ variations at various gate voltage *V*_G_ of c) a second-order nonlinear equation task and d) a NARMA2 task, respectively, in a magnetic field of 186 mT. e) NMSE as a function of the number of nodes on the second-order nonlinear equation task. f) NMSE_var._ as a function of the number of nodes on the NAMRA2 prediction task.

**S4. The number of nodes dependence of the errors of second-order nonlinear dynamic equation tasks and NARMA2 tasks**

**Figures S5**a and b show the number of nodes dependent on NMSE and NMSE_var._ of a second-order nonlinear dynamic equation task and an NARMA2 task, in magnetic fields of 169 mT and 186 mT, repectively. For all conditions, both errors reduce monotonically as the number of nodes increases. Except for the 2 ns pulse interval, there is a tendency for both NMSE and NMSE_var._ to decrease as the pulse interval is shortened. Then, NMSE and NMSE_var._ reach minimum values of 6.41 × 10^-4^ and 9.53 × 10^-3^ at an interval of 5 ns. As shown in Figures S5c and d, the errors with a magnetic field of 186 mT show the same tendency compared to those with a magnetic field of 169 mT, and reach values of 6.91 × 10^-5^ and 1.23 × 10^-2^. Thus, the best condition for those tasks is a pulse interval of 5ns.


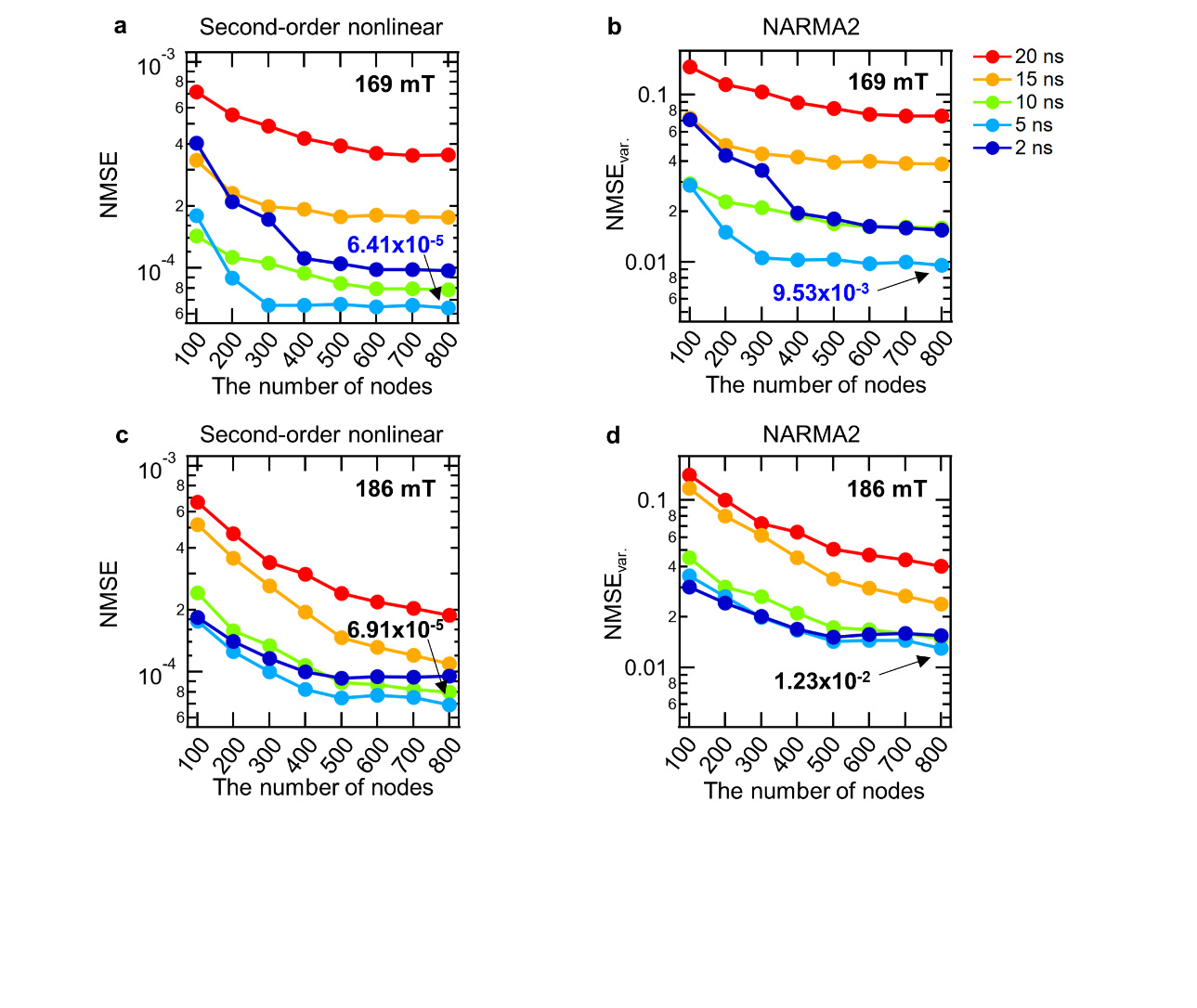


**Figure S5.** The result of time-series processing tasks. NMSE and NMSE_var._ as a function of the number of nodes of a) a second-order nonlinear equation task and b) a NARMA2 task, respectively, in a magnetic field of 169 mT. NMSE and NMSE_var._ as a function of the number of nodes of c) a second-order nonlinear equation task and d) a NARMA2 task, respectively, in a magnetic field of 186 mT.

**S5. Micromagnetic simulation of the nonlinearity variation of the interfered spin wave at various *V*_G_**

To investigate nonlinearity variation at various *V*_G_, we performed a theoretical simulation using a Mumax3 micromagnetic simulator.^[S5]^ YIG measuring 380 μm × 90 μm × 0.12 μm was used for the spin wave waveguide to investigate the spin dynamics near a surface region in the vicinity of an antenna. The two exciters used consisted of a signal line (10 μm × 90 μm × 0.12 μm) and two ground lines (20 μm × 90 μm × 0.12 μm). The detection areas of the two detectors are 10 μm × 90 μm × 0.12 μm, which corresponds to the signal lines of the detectors. The mesh was cubic, measuring 40 nm × 40 nm × 40 nm along the Cartesian coordinates defined by an origin at the center of the surface plane on the YIG. A spin with a saturation magnetization of 157.9 kA/m was located at every mesh corner. The simulation time step was 10 ps. The material parameters estimated from experimental measurement at *V*_G_ = 0.0 V, 0.2 V, 1.6 V, and 2.8 V are a saturation magnetization of 157.9 kA/m, 153.5 kA/m, 148.3 kA/m, and 148.5 kA/m, a uniaxial anisotropy along the z-axis *K*_U_ of -6.32 kJ/m^3^, -5.94 kJ/m^3^, -5.73 kJ/m^3^, and -5.75 kJ/m^3^. To simulate spin wave property variation at various *V*_G_, these parameters were set to various values that corresponded to the experimental result shown in Figure 1e. A cubic magneto-crystalline anisotropy of 0.0, an exchange stiffness constant of 3.7 pJ/m, and a damping constant of 1 × 10^-4^ were used as typical values of the YIG. A static magnetic field of 0.3 T was applied along the z-axis (i.e., perpendicular to the YIG surface) over the entire region. An excitation field, with a rectangular shape-pulse, was set at 80 mT along the y-axis at the exciters applied in the exciter. The field is estimated as a simulation result shown in the literature.^[S2]^ The pulse interval was set to 5 ns, which is the best condition for time-series data prediction tasks. The field vectors at the signal and ground lines were positive and negative, respectively, since electric current flows in the opposite direction.

Micromagnetic simulations of the interfered spin wave at various *V*_G_ was performed to reveal the origin of the highest performance achieved at *V*_G_ of 1.6 V. The *V*_G_ dependence on the nonlinearity of an interfered spin wave was investigated by comparing waveforms simulated under various *V*_G_ conditions. Pulse signals were repeatedly input to intermittently excite spin waves. Spin waves, excited at intervals with the same conditions as in the experiment, were excited intermittently, thereby introducing nonlinearities due to interference and history effects. Nonlinear interference from simulations has also been reported in theoretical spin wave reservoir computing.^[S6]^ **Figure S6**a shows the simulation model of a nonlinear interfered spin wave multi-detection. The size of the antennas and the distance between them were set to the same dimensions as in the actual device. Figures S6b-i show the simulated spin wave motion with an external magnetic field of 0.3 T, an input pulse interval of 5 ns, and *V*_G_ = 0.0 V. The input signal at the position corresponding to the GND line is shown in Figure S6j. As can be seen in Figures S6b and d, the spin waves excited at two exciters show different waveforms, even though the same detector is used. This result is in good agreement with the behavior observed in the experimental result. The nonlinear interference of spin waves was also observed in the simulation since there is a finite difference *Δm_x_*, which is subtraction of the interfered spin waves excited by Exciter A and Exciter B (Figure S6f) and the linear summation of the two spin waves excited by Exciter A (Figure S6b) and Exciter B (Figure S6d), as shown in Figure S6h. This proves that spin waves interfere nonlinearly due to magnetic dipole interactions between spin waves. A similar relationship was confirmed in the case of spin waves at Detector B, as shown in Figures S6c, e, g, and i. The nonlinear ratio variation of interfered spin waves at various *V*_G_ is shown in Figure S6k. The nonlinearity ratio at Detector A and Detector B shows a top-convex plot. It reaches maximum values of 112 and 138 % at *V*_G_ of 1.6 V. This result is in good agreement with the experimental result, with the lowest NMSE and NMSE_var._ for second-order nonlinear dynamic equation and NARMA2 tasks achieved at a *V*_G_ of 1.6 V. Thus, it was possible to theroretically confirm that the condition *V*_G_ = 1.6 V gives the system the highest nonlinearity.

**Figure S6.** The simulated spin wave motion and its nonlinear interference. a) Simulation model of nonlinear interfered spin wave multi-detection with a waveguide of 380 × 90 × 0.12 μm^3^. The cubic mesh employed measured 40 nm × 40 nm × 40 nm. Simulated spin wave motions, which are excited at b) Exciter A, d) Exciter B, and f) Exciter A and Exciter B, at Detector A. Simulated spin wave motions that are excited at c) Exciter A, e) Exciter B, and g) Exciter A and Exciter B, at Detector B. The difference between the interfered spin wave and the linear summation at h) Detector A and i) Detector B. j) Magnetic field generated at Exciter A and Exciter B. k) Nonlinear ratio variation interfered spin wave at various pulse intervals.


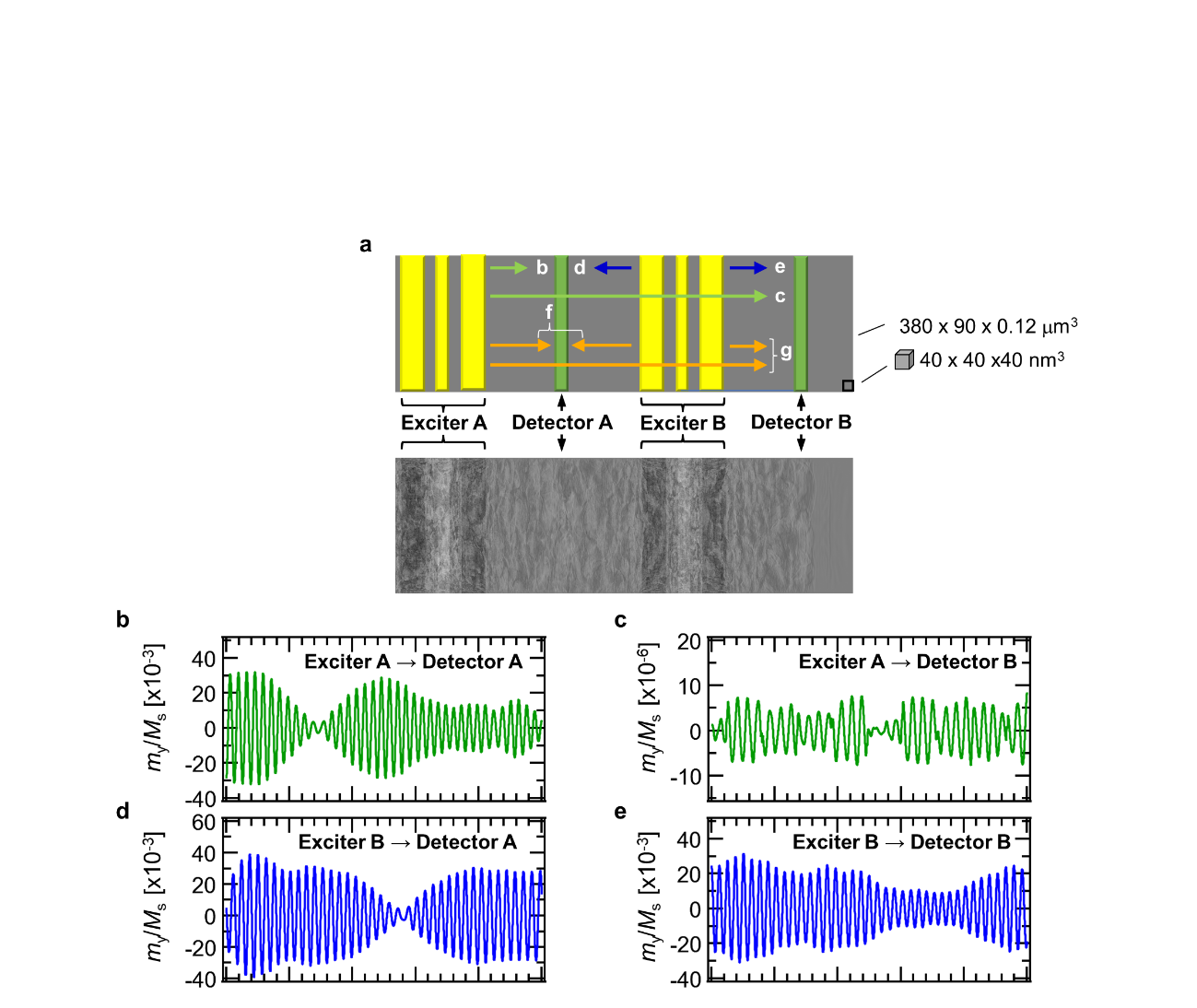

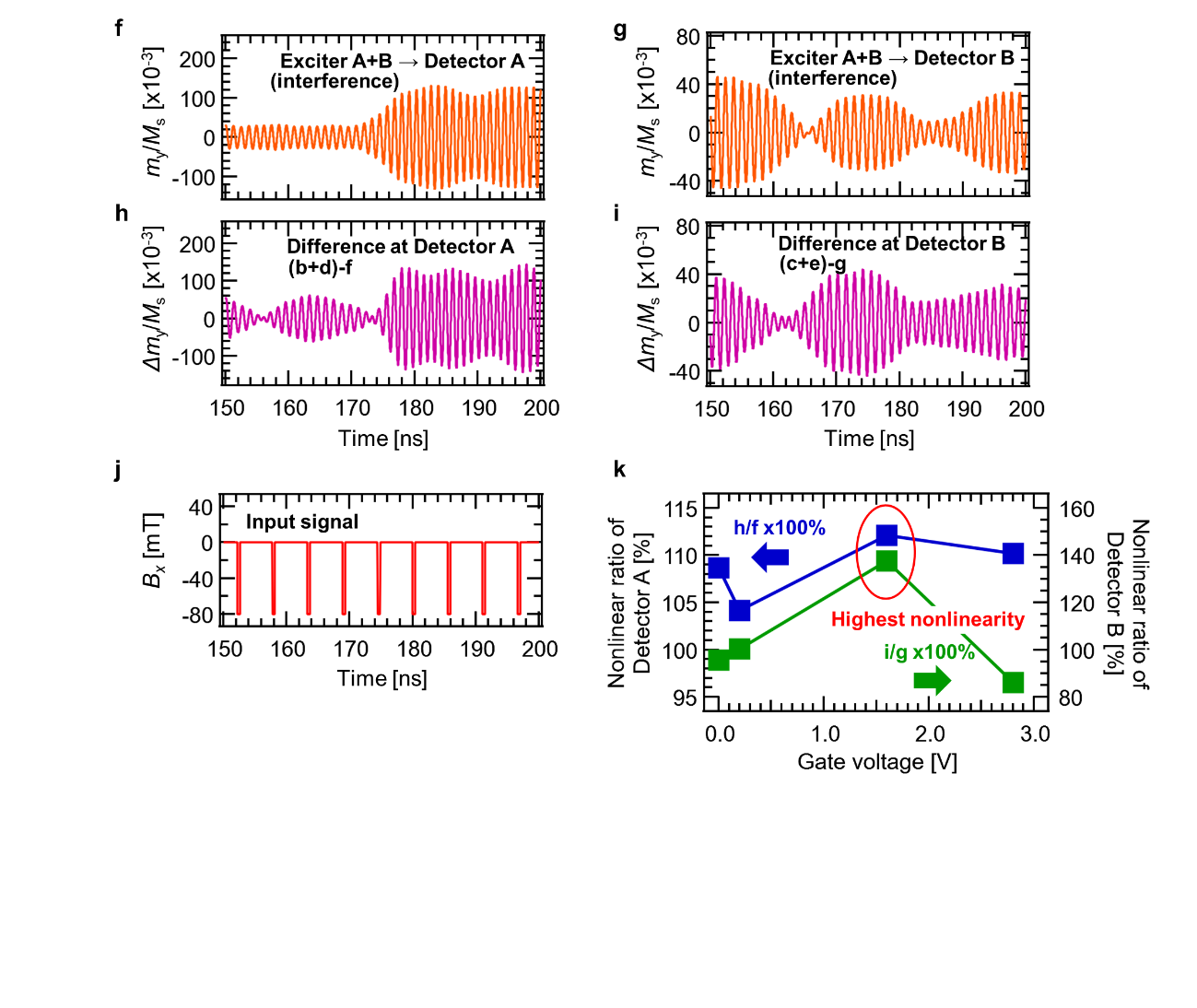


**S6. Ridge parameter dependence of the computational performance of the iono-magnonic reservoir**

To prevent overfitting in reservoir computing, the input data should be large relative to the number of nodes in the reservoir. This is equivalent to making the data length sufficiently long for training time series data. In this study, training waveforms with a data length of 3500 are used, and the number of nodes in the reservoir is 800, thus satisfying the requirement for a more significant number of data than of nodes.

Whether the network is overfitted or not can be determined by examining the ridge parameter *β* dependence on the computational performance of the iono-magnonic reservoir. This study uses linear regression (i.e., *β* = 0.0) to train the network. In the case of linear regression, learning is achieved by minimizing the error between the target value during training and the output value of the reservoir. Suppose the output weights are increased to minimize the error. In that case, the network becomes applicable only to training, and overfitting occurs when the network loses generalization performance that can be applied to test data. Ridge regression is a method for suppressing the overfitting of a network and training is done by minimizing the cost function. On the other hand, by introducing the product of the ridge parameter and the sum of squares of the readout weights into the cost function, the weights tend to be smaller than in linear regression, and overlearning for the training waveform can be suppressed. As shown in **Figures S7**a and b, increasing the ridge parameter *β* increased the NMSE during training for both the second-order nonlinear equation task and the NARMA2 prediction task, and decreased the NMSE in the test course. This indicates that the generalization performance of the network was improved. In the range where *β* is small, the NMSE in training decreased and the NMSE in testing increased slightly. This implies a slight deterioration in the generalization performance of the network. However, the generalization performance of the network to obtain sufficient computational performance is retained, since the features of the target waveform in the test are well captured and the NMSE is very low.

Therefore, although overlearning was observed in the training of the iono-magnonic reservoir network using the linear regression, the generalization performance was maintained and high performance was achieved using linear regression, which is computationally less expensive. Furthermore, ridge regression is computationally expensive compared to linear regression due to the large number of terms in the cost function. Therefore, training the network with linear regression, which is computationally less expensive in this study, is an advantageous method in terms of implementation.

The dependence of the computational error on *β* shows that the lowest NMSE and NMSE_var._ is obtained when *β* = 0.1, with an NMSE of 5.88 × 10^-5^ for the second-order nonlinear equation task and an NMSE_var._ of 8.67 × 10^-3^ for the NARMA2 prediction task. The NMSE_var._ in the NARMA2 prediction task was 8.67 × 10^-3^.


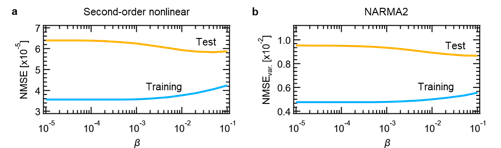


**Figure S7**. a) The ridge parameter *β* dependence of NMSE on a second-order nonlinear equation task. b)The *β* dependence of NMSE_var._ on a NARMA2 task.

**S7. Difference in computational performance by data shuffling**

To investigate whether the method of taking out the test and training periods affects computational performance, two patterns were tested. **Figure S8**a shows illustrations of a training and testing data set. The first pattern (i.e., Pattern A) is the method in which the NARMA task was performed, with 3500 of the first 4000 steps of the 5000 step long time series data as the training period, and a test period consisting of 500 steps out of the second 1000 steps. The first 500 steps of the training and test periods were discarded. On the other hand, in the second pattern B, 500 of the first half of 1000 steps of the 5000-step long time series were used as the test period, and 3500 of the second half of 4000 steps were used as the training period. As shown in Figure S8b, in Pattern B, the NMSE worsened slightly to 6.84 × 10^-5^ for the second-order nonlinear transformation task, while the NMSE_var._ was reduced to 7.30 × 10^-3^ for the NARMA2 prediction task, and computational performance was improved only in the case of the NARMA2 prediction task.


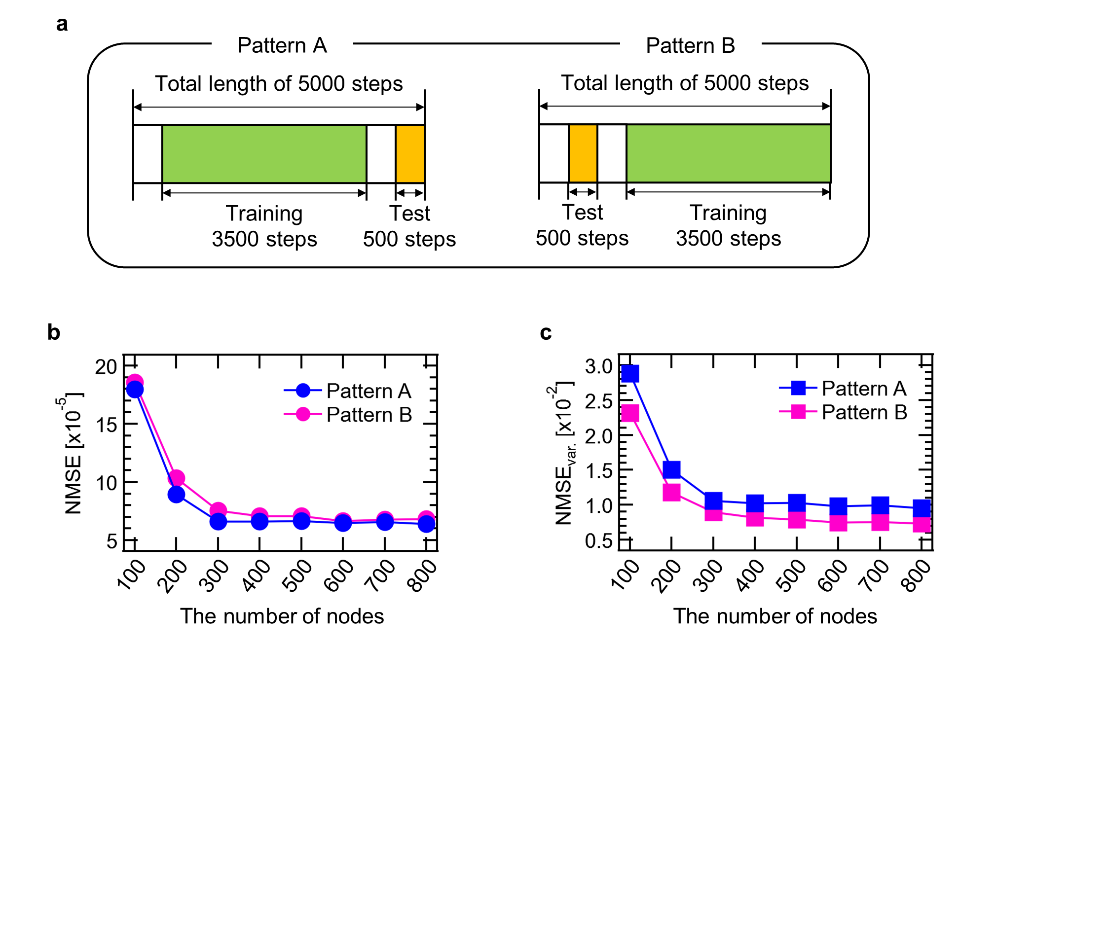


**Figure S8.** a) Illustrations of a training and testing data set. The total length of the time step is 5000. Pattern A (Pattern B) was divided into the first half, 3500 steps (500 steps) for training (testing), and the second half, 500 steps (3500 steps) for testing (training). A white region with 500 steps was discarded. b) Number of nodes dependent on NMSE for a second-order nonlinear equation task. c) Number of nodes dependence of NMSE_var._ for a NARMA2 task.

**S8. The reliability and reproducibility of the iono-magnonic reservoir**

The reliability/reproducibility of the iono-magnonic reservoir was verified. *V*_G_ = 0.0 V and 1.6 V were alternately applied for 1800 s each, and a pulsed random wave was input at *V*_G_ = 0.0 V to acquire the spin wave signal. A magnetic field of 169 mT was applied, and the pulse interval was 5 ns. The NMSE_var._ of the NARMA2 task, using the acquired signal, is shown in **Figure S9**a**.** The slight variation in plots results from the different proton content of YIG at *V*_G_ = 0.0 V due to historic effect during the application of *V*_G_ = 1.6 V. However, as represented as a dashed line, no significant change in NMSE_var._ was observed with increasing number.

As shown in Figure S9b, spin wave signals, which are measured at *V*_G_ = 0.0 V and used for solving the NARMA2 task shown in Figure S9a, exhibit no significant change over cycle measurement. Although there is a slight decrease in spin wave signals with each increase in the number of cycles, due to relatively strong nonvolatility, it was confirmed that this decrease does not affect the computational performance, as shown in Figure S9a.

Note that strictly reversible ion-gating is not necessary in this physical reservoir. The reason for reversibility not being necessary in this physical reservoir is that it can, in advance, process the input data with parallel eight iono-magnonic devices under various *V*_G_ applications (i.e., 0.0, 0.2, 1.6, 2.0, 2.2, 2.4, 2.6, and 2.8 V). This approach solves the problem of requiring both strict reversibility of the physical device in physical reservoir computing and a long *V*_G_ application time of 1800 seconds, which was needed in this study. Improving reversibility in the iono-magnonic device, in terms of the ferromagnetic and electrolyte materials selected, will be the subject of future work.


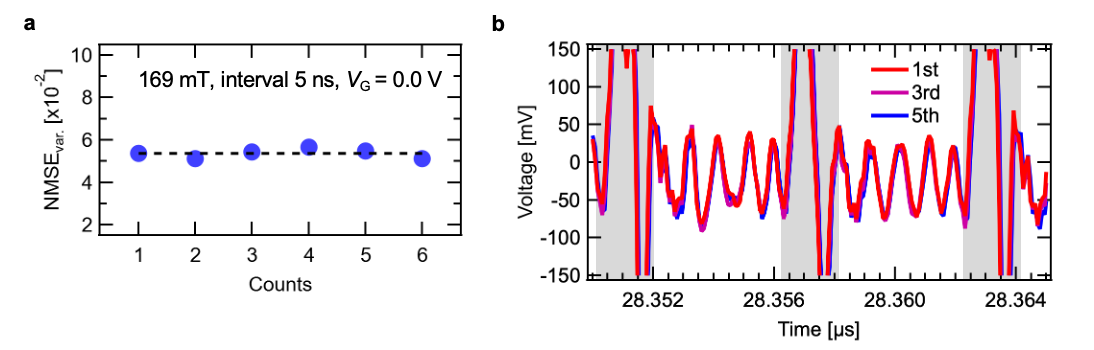


**Figure S9.** a) Repeatability of the iono-magnonic reservoir for a NARMA2 task at 169 mT, with an interval of 5 ns and *V*_G_ = 0.0 V. b) Spin wave signals of the iono-magnonic reservoir at 1st, 3rd, and 5th cycles under *V*_G_ = 0.0 V and 169 mT applications.

**S9. The number of nodes dependence of the errors of Mackey-Glass chaotic time-series prediction task**

**Figures S10**a and b show MSE variations at various gate voltage *V*_G_ application in a magnetic field of 169 mT and 186 mT, respectively. MSEs decreases as the pulse interval becomes shorter, with the lowest MSE (3.20 × 10^-3^) achieved when using a magnetic field of 169 mT, a *V*_G_ of 2.2 V, a pulse interval of 5 ns. This optimized condition for the Mackey-Glass time-series prediction task differs from that for the second-order nonlinear equation and NARMA2 tasks. The Mackey-Glass time series prediction task uses time series data generated from past memories and differential equations containing nonlinear terms, as described in equation 4. Such autonomous time series data evolving from its own past states are input to a physical device, which processes and predicts the time series data, using its ability to map nonlinearly in higher dimensional space. The characteristics of the input time-series data may have led to differences in the optimal voltage conditions from the case of the NARMA2 task (169 mT, 5 ns interval, and *V*_G_ = 2.2 V). The optimal condition for this task (169 mT, 5 ns interval, and *V*_G_ = 2.2 V) had relatively large memory capacity and high complexity, leading to the highly accurate prediction of the chaotic time series data. The memory capacity and complexity are shown later in this report. Figure S10c shows the number of nodes dependence of MSE and RMSE of the Mackey-Glass chaotic time-series prediction task on the optimal condition. The errors were successfully reduced as the number of nodes increased, until the errors reached their lowest value (i.e, MSE of 3.2 × 10^-4^ and RMSE of 5.66 × 10^-4^). As shown in Figures S10d and e, the MSE decreased monotonically as the number of nodes increased. When a shorter interval was used, the MSE decreased. Each voltage state in the short interval had a smaller error, as shown in Figures S10a and b. Thus, the reservoir state was generated as a population of voltage states with high expressive power, and a higher dimension by voltage state was successfully achieved. In all *V*_G_ application states, the MSE was smaller at 169 mT than at 186 mT, with a minimum MSE of 1.08 × 10^-3^ at an interval of 5 ns, with 800 nodes.


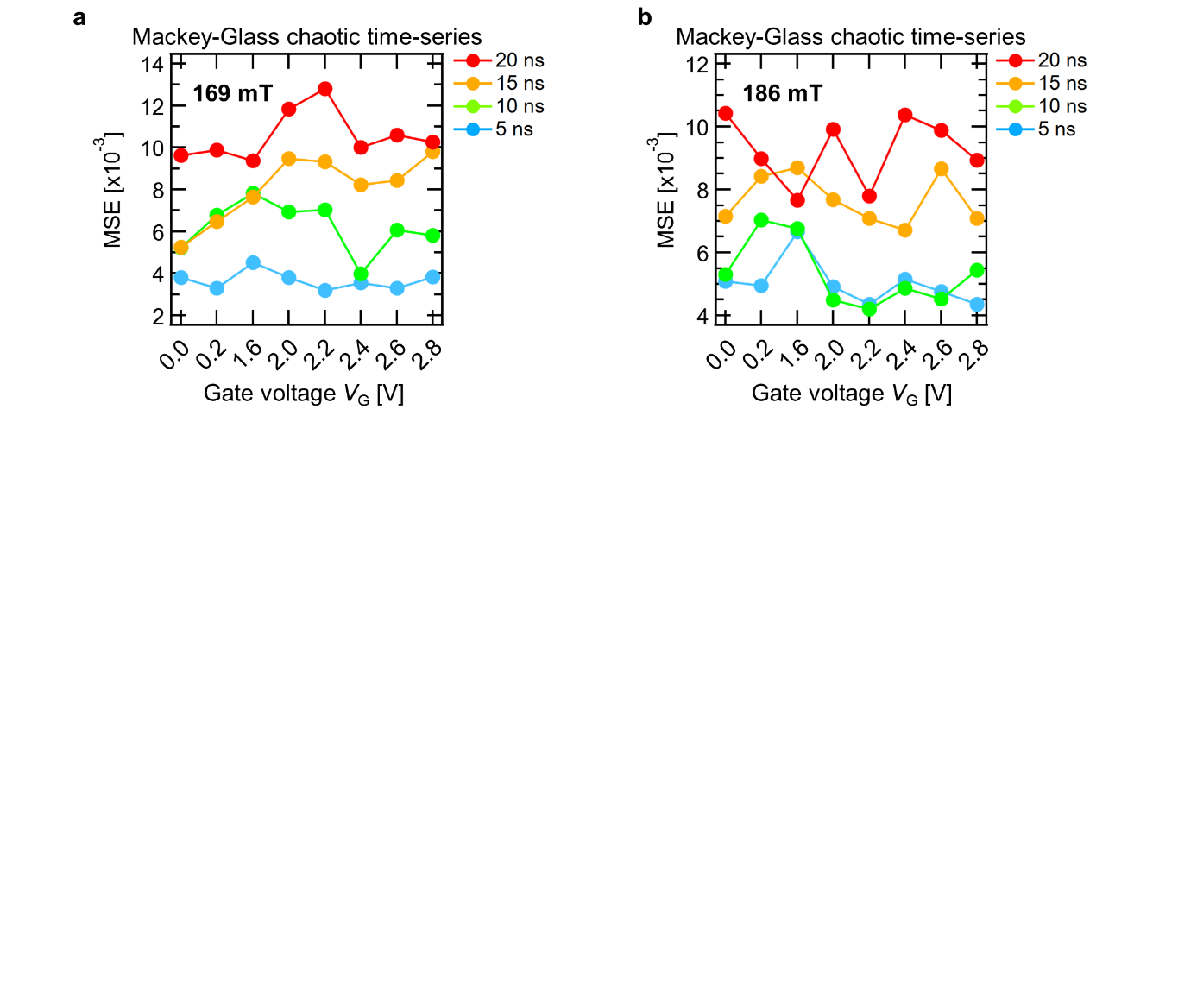

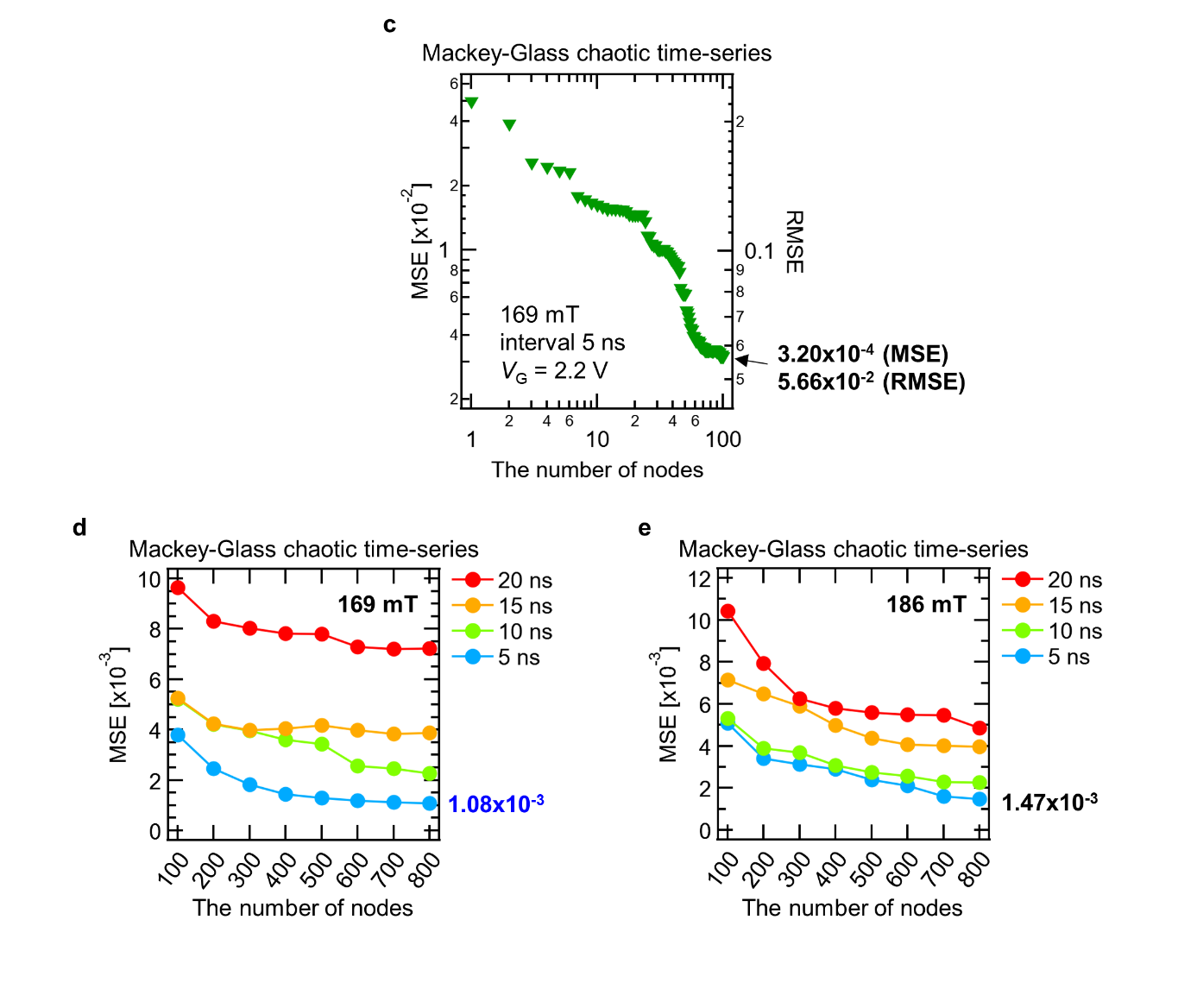


**Figure S10.** Results of Mackey-Glass chaotic time-series prediction tasks. a),b) MSE variation at various *V*_G_ application in magnetic fields of 169 mT and 186 mT. c) MSE and RMSE as a function of the number of nodes on the Mackey-Glass chaotic time-series prediction task. d),e) MSE as a function of the number of nodes in magnetic fields of 169 mT and 186 mT.

**S10. The ability to map in a higher dimensional space and nonlinearity at various *V*_G_ in the iono-magnonic reservoir**

**Figures S11**a-h show the non-correlation coefficients (*A_ij_*) between *X_i_* and *X_j_* at various *V*_G_ in a magnetic field of 169 mT. Here, *A_ij_* is defined as follows;^[S7-S9]^

$$A_{ij}=1-r_{ij}=1-\frac{\mathrm{Cov}\left( X_{i},X_{j} \right)}{\mathrm{Var}\left( X_{i} \right)\mathrm{Var}\left( X_{j} \right)}, (S3)$$

where *r_ij_* is the correlation constant and Cov(*X_i_*, *X*_j_) is the covariance between vectors *X_i_* and *X_j_*, and Var(*X*) ≅ Cov(*X*, *X*). Thus, there is a strong variation between *X_i_* and *X_j_* when *A_ij_* is close to 1 (i.e., high ability to map in the higher dimensional space), while there is no variation between *X_i_* and *X_j_* when *A_ij_* is near 0 (i.e., low ability to map in the higher dimensional space). Note that the diagonal components (*A_ii_*) in Figures S11a-h are 0.0 since *A_ij_* is 0.0 when *i* = *j*. In all conditions, *i* and *j* in ranges 5 -10 and 45 - 50 show an *A_ij_* of 1.0. While most of the heatmap shows green and yellow, indicating some correlation, there is the tendency for *A_ij_* to be high in ranges *X*_5_-*X*_10_, *X*_45_-*X*_50_, and *X*_85_-*X*_90_ and their vicinity. These nodes do not correlate with the others, and strongly contribute to the ability of the reservoir to map in high-dimensional feature space. The same result is observed in every *V*_G_ condition. The summation variation of such *A_ij_* at various *V*_G_ is shown in Figure S11i. While the summation of *A_ij_* does not depend on *V*_G_ in either magnetic field, its value at 169 mT is higher than at 186 mT, indicating that the ability to map in the higher dimensional space at a value of 169 mT is superior to that at 186 mT. The nonlinearity of the reservoir is evaluated from a maximum Lyapunov exponent λ_max_, which is an indicator of nonlinear (i.e., a chaotic state) or linear (i.e., an ordered state). As shown in Figure S11j, λ_max_ in a magnetic field of 169 mT is lower than that in a 186 mT magnetic field. This result shows that the reservoir state in a magnetic field of 169 mT exhibits strong nonlinearity with positive λ_max_ due to chaotic behavior, although the value is relatively small compared to those under 186 mT. The return map with *X_i_*(*k*) versus *X_i_*(*k*+1) obtained from the reservoir states of nodes 4 (*i* = 4), with various *V*_G_ applications, is shown in Figures S11k-r. The trajectory with finite width indicates that the reservoir state has a relatively unstable response (i.e., chaos) that varies slightly from period to period. The trajectory at *V*_G_ = 0.0 V differs from the other since λ_max_ shows a relatively large value, indicating that the reservoir state at *V*_G_ = 0.0 V has a stronger chaotic state. From the results shown in Figures S11i and j, the iono-magnonic reservoir shows high reservoir performance for the ability to map in the higher dimensional space and nonlinearity in a magnetic field of 169 mT.


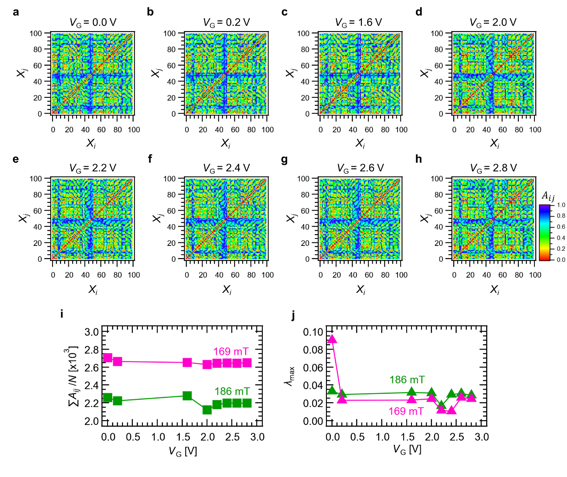

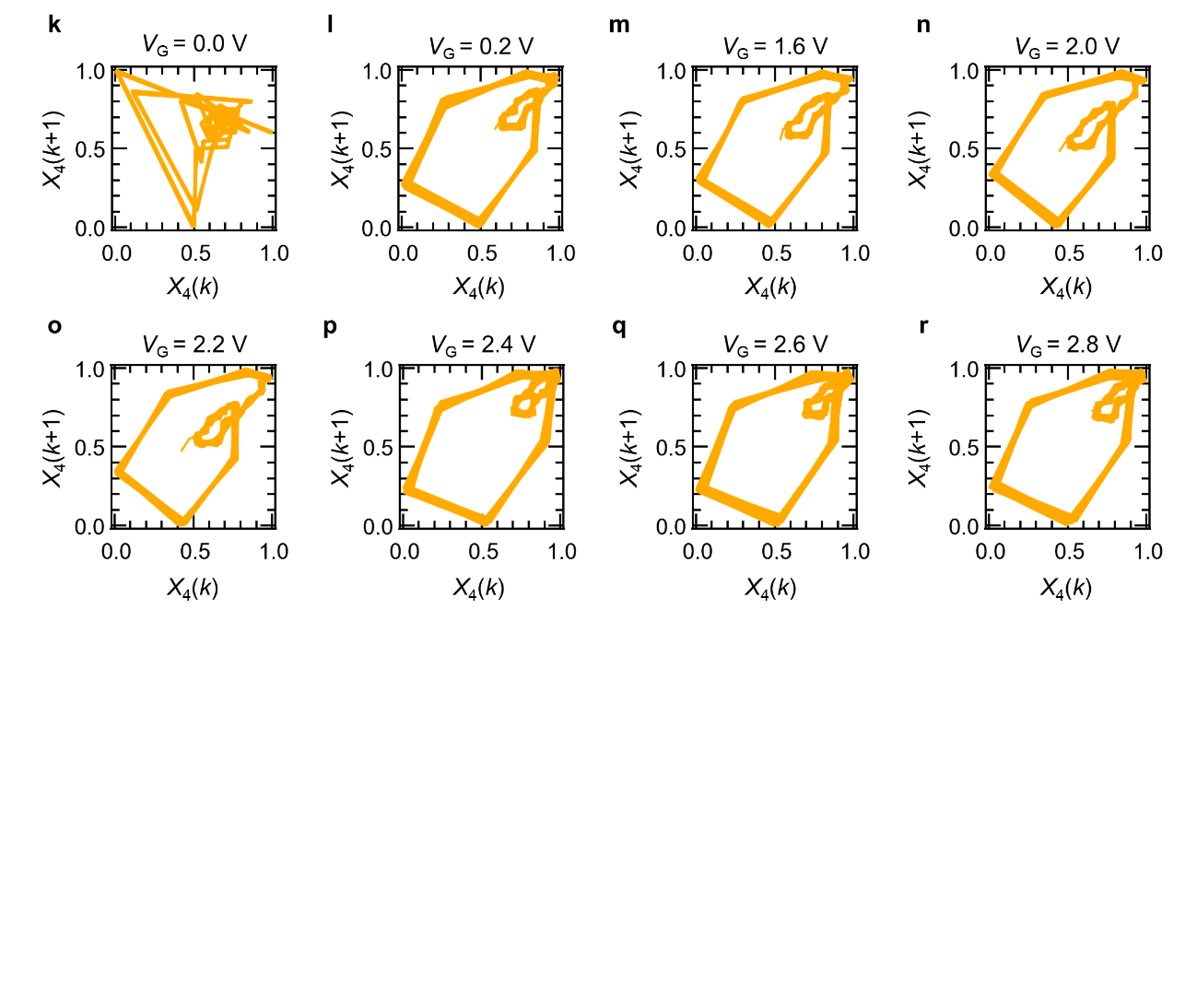


**Figure S11.** Evaluation of the ability to map in high dimensional space, and the nonlinearity of the iono-magnonic reservoir. Non-correlation coefficient (*A_ij_*) heatmaps of the iono-magnonic reservoir with a) *V*_G_ = 0.0 V (100 nodes), b) *V*_G_ = 0.2 V (100 nodes), c) *V*_G_ = 1.6 V (100 nodes), d) *V*_G_ = 2.0 V (100 nodes), e) *V*_G_ = 2.2 V (100 nodes), f) *V*_G_ = 2.4 V (100 nodes), g) *V*_G_ = 2.6 V (100 nodes), h) *V*_G_ = 2.8 V (100 nodes). i) The summation of *A_ij_* normalized by the number of nodes *N* as a function of *V*_G_. j) The Maximum Lyapunov exponent λ_max_ as a function of *V*_G_. Return maps in *X*_4_(*k*) versus *X*_4_(*k*+1) at k) *V*_G_ = 0.0 V, l) *V*_G_ = 0.2 V, m) at *V*_G_ = 1.6 V, n) *V*_G_ = 2.0 V, o) *V*_G_ = 2.2 V, p) *V*_G_ = 2.4 V, q) *V*_G_ = 2.6 V, r) *V*_G_ = 2.8 V.

**S11. Evaluation of the complexity and permutation entropy of the reservoir state of the iono-magnonic reservoir**

Since chaos is not random but is comprised of complex behaviors, complexity (*C*) and permutation entropy (*H*_e_) were introduced to evaluate whether the feature of time-series data is complexity or randomness.^[S10-S13]^ **Figures S12**a-h show *C* of the reservoir states of the iono-magnonic reservoir and various time-series data as a function of *H*_e_. There are the minimum and maximum values of *C* (i.e., *C*_min_ and *C*_max_), and the plot distribution, provides visual support for determining the characteristics of time-series data (i.e., randomness, noisiness, periodicity, and chaos). When the signal is completely random (i.e., white noise), *H*_e_ and *C* are 1.0 and 0.0, respectively. Fractional Brownian motion (fBm), which is chromatic noise, is a stochastic process,^[S11]^ and its complexity *C* and entropy *H*_e_ depend on Hurst exponent *H*. Thus, it can be judged whether the behavior of time-series data is deterministic or stochastic, since the curve drawn by these plots is an important indicator expressing a feature of time-series data. Concretely speaking, a signal is chaotic (stochastic) when a plot locates above (below) the fBm curve. For example, *C* and *H*_e_ of the logistic map, which is known as a representative example of chaos, increase as coefficient *r* increases, and locates above the curve of chromatic noise.^[S11]^ The logistic map is described as follows,

$f\left( t+1 \right)=rf(t)(1-f\left( t \right))$ (S4).

Its behavior largely changes depending on parameter *r*. The logistic map is deterministic chaos by simple nonlinear mapping, and its *C* closes to maximum value when *H*_e_ has a medium value, as shown in Figures S12a-h. If a signal shows periodic behavior, the combination of *H*_e_ and *C* is within the region of 0.1~0.2. Based on the above relationship, we evaluated permutation entropy *H*_e_ dependence of complexity *C* at various *V*_G_. Although parabolic-shaped distributions of the plots can be seen in most *V*_G_ conditions, the plots at *V*_G_ = 0.0 V are condensed. The combinations of *C* and *H*_e_ of all nodes were plotted in the chaos region located above the fBm curve. Therefore, it can be said that the dynamics seem to be chaotic.


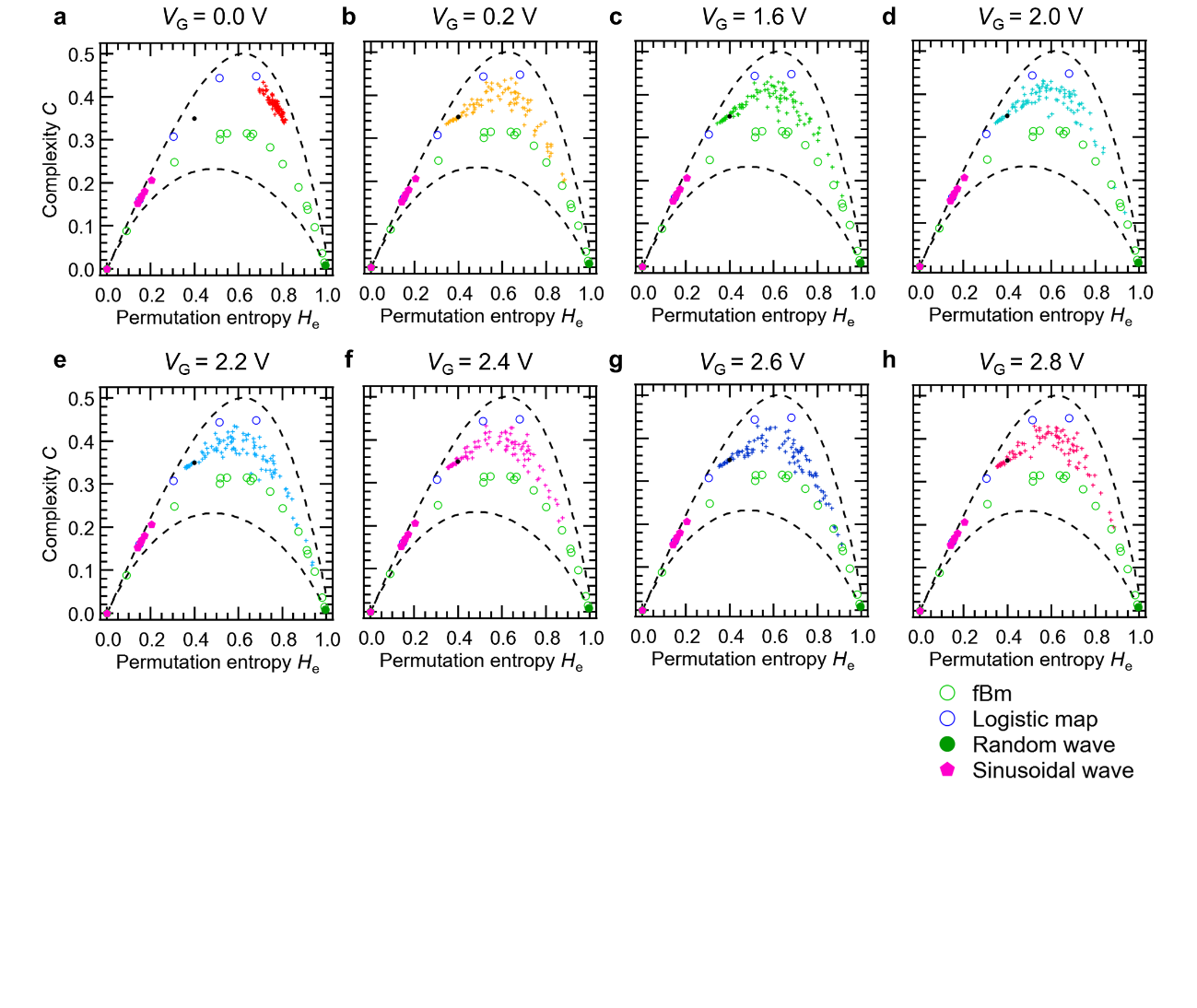


**Figure S12.** Evaluation of complexity *C* variation at various permutation entropy *H*_e_ in a magnetic field of 169 mT with a pulse interval of 5 ns. The distribution of *C* and *H*_e_ at a) *V*_G_ = 0.0 V, b) *V*_G_ = 0.2 V, c) *V*_G_ = 1.6 V, d) *V*_G_ = 2.0 V, e) *V*_G_ = 2.2 V, f) *V*_G_ = 2.4 V, g) *V*_G_ = 2.6 V, h) *V*_G_ = 2.8 V. Also shown are plots of the fractional Brown motion (fBm), logistic map, random wave, and sinusoidal wave. The lower and upper dashed lines represent the lower and upper limits of *C*.

**Figures S13**a-f show *C* variation at various pulse intervals of 2-20 ns at 169 mT. A parabola-shaped distribution can be seen in all conditions. The distribution shifts to the high *H*_e_ side with increased pulse intervals, and some plots show random behavior since such *H*_e_ is approximately 1.0 above a 10 ns pulse interval. This result means that the effective nodes in the reservoir is decreased due to an increase in the nodes that do not contribute to the role of the reservoir (i.e., nonlinear transformation) due to their random behavior. Thus, the computational performance at pulse intervals above 10 ns is relatively low, compared with shorter pulse intervals. At 186 mT, a distribution of *C* variation at various pulse intervals was observed, as shown in **Figures S14**a-f.


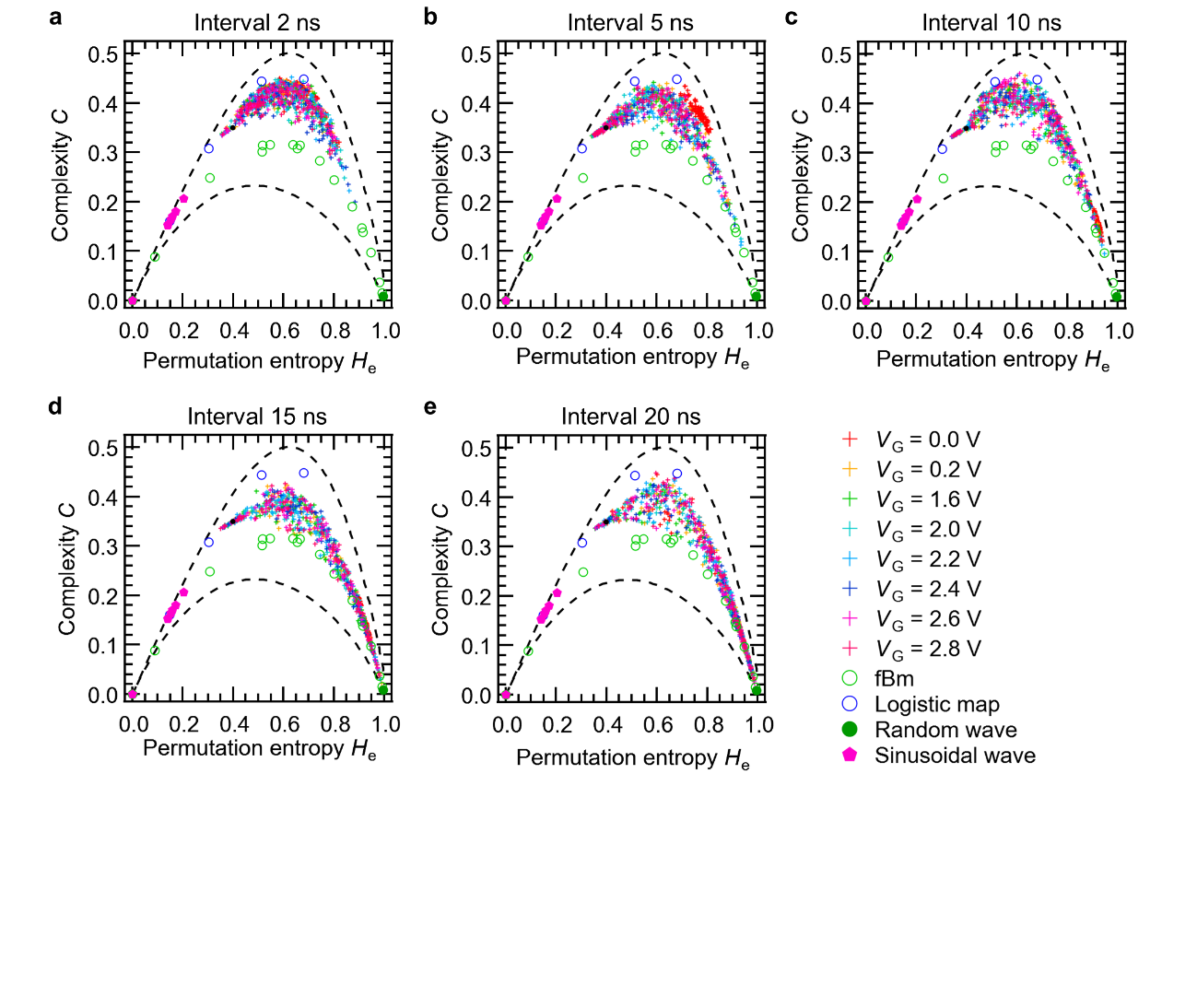


**Figure S13.** Evaluation of complexity *C* variation at various permutation entropy *H*_e_ in a magnetic field of 169 mT. The distribution of *C* and *H*_e_ at intervals of a) 2 ns, b) 5 ns, c) 10 ns, d) 15 ns, and e) 20 ns. Also shown are plots of the fractional Brown motion (fBm), logistic map, random wave, and sinusoidal wave. The lower and upper dashed lines represent the lower and upper limits of *C*.


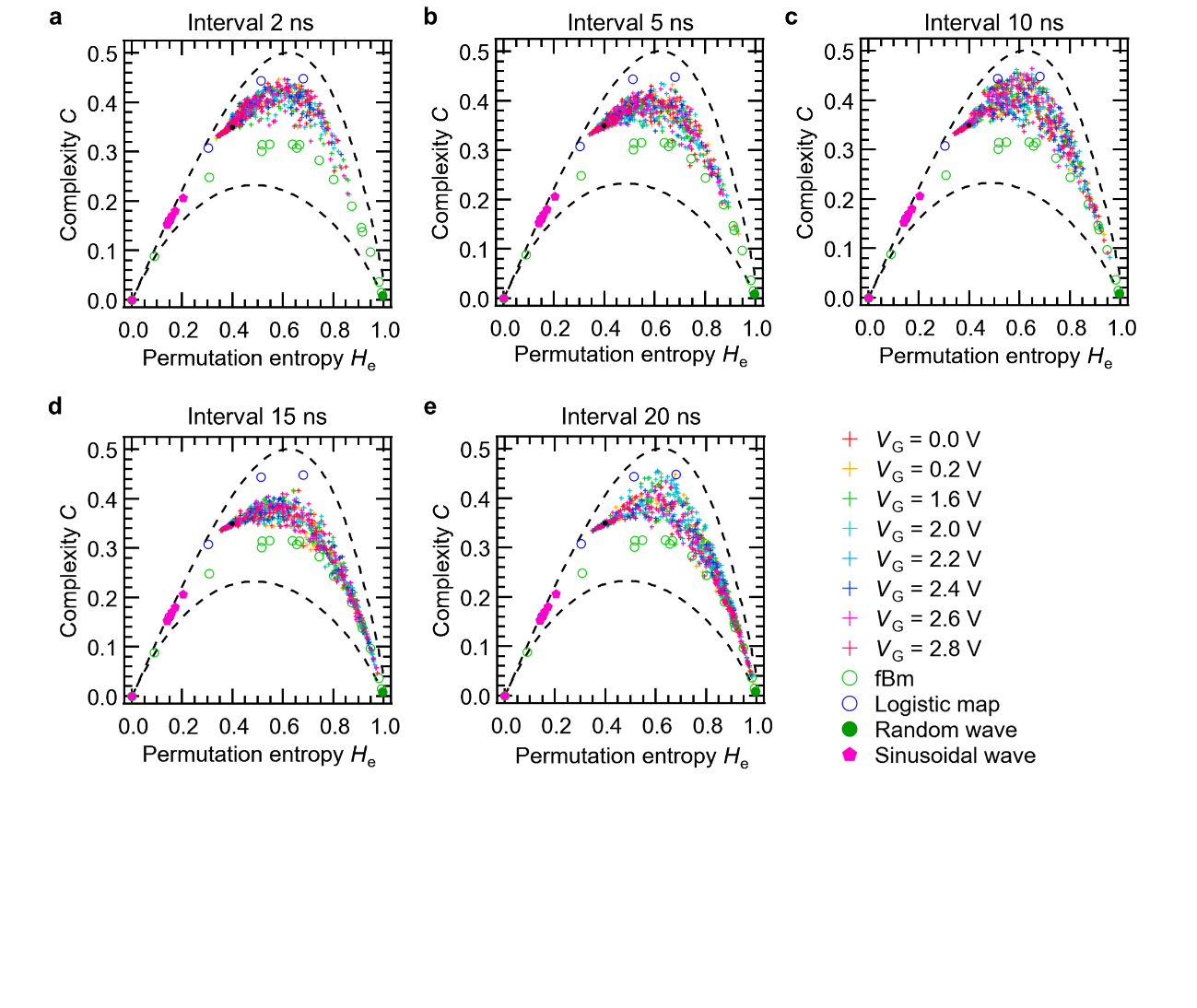


**Figure S14.** Evaluation of complexity *C* variation at various permutation entropy *H*_e_ with a magnetic field of 186 mT. The distribution of *C* and *H*_e_ at intervals of a) 2 ns, b) 5 ns, c) 10 ns, d) 15 ns, and e) 20 ns. Also shown are plots of the fractional Brown motion (fBm), logistic map, random wave, and sinusoidal wave. The lower and upper dashed lines represent the lower and upper limits of *C*.

When the time interval between virtual nodes is short with respect to the dynamics time scale, the node connections in the reservoir are strong (i.e., dense). Conversely, when the time interval between virtual nodes is long with respect to the dynamics time scale, the node connections in the reservoir are weak and the self-coupling contribution of each node is strong (i.e., sparse). All of which means that the reservoir states are different when the time scale of the input is varied with respect to the dynamics occurring in the physical device.

Since the virtual nodes in this study are taken from a detected transient response (i.e., a spin wave), they are coupled in the time scale.^[S14]^ The virtual nodes are taken from a region corresponding to the interval of the pulsed input signal. For input condition optimization, the interval ranges from 5 ns-20 ns for each condition, and the virtual nodes are taken out from an entire region corresponding to the interval. In other words, the temporal coupling between virtual nodes becomes weaker (stronger) as the pulse interval becomes longer (shorter), and the coupling strength between nodes in the reservoir layer changes with the input interval. This is consistent with the example reported by Appeltant et al.^[S14]^

A spectral radius, which is the largest absolute value of the eigenvalues of the weight vector in the reservoir layer of an echo-state network (ESN), is known as an indicator of the nonlinearity of the reservoir layer.^[S15]^ Change in the weights of the reservoir layer correspond to change in the spectral radius. When the connection between nodes is strong, the weights are larger and the spectral radius is larger, resulting in larger nonlinearity in the reservoir, which makes the nonlinearity of the reservoir stronger. Furthermore, when the spectral radius exceeds a certain threshold value, it behaves chaotically. On the other hand, when the connection between nodes is weak, the weights become small. Then, the spectral radius also becomes small, and the reservoir becomes more linear. Since the relationship between the weights matrix and the spectral radius is clarified only in a typical echo-state network (ESN),^[S15]^ the one between the weights connecting the virtual nodes and the spectral radius in the physical reservoir have not yet been clarified. Although it is difficult at present to estimate the specific weights in the physical reservoir, it is possible that the relationship reported by Appeltant et al., between input scaling and the weights as discussed above, could be extended to spectral radius and nonlinearity by close analysis of the dynamical behavior of the physical reservoir, so as to clarify its mapping function. It would be a very useful guideline for the future development of physical reservoirs.

**S12. *V*_G_ dependence of the computational peformance in the iono-magnonic reservoir**

A short-term memory task, which is widely used to evaluate the performance of physical reservoirs, was performed.^[S1]^ A plot of the memory capacity at each *V*_G_ is shown in **Figure S15**. Application of *V*_G_ = 1.6 V yielded a good memory capacity, and the *V*_G_ dependence of the memory capacity was similar to that of the computational performance (i.e., the inverse proportion of NMSE) observed in the iono-magnonic reservoir. This indicates that, in addition to the nonlinearity of the system, a large memory capacity contributes to high computational performance.

In addition, as already shown in the simulation, there is strong nonlinearity at 1.6 V compared to other voltage states. Furthermore, spin waves are excited at the rising and falling edges of the input pulse waveform. The excitation frequency of the spin wave at 169 mT, which was the optimal condition for the task, is close to the frequency of the input pulse signal, and results in excellent short-term memory and nonlinearity. The pulse frequency of the input signal was fixed, although the interval of the pulse signal was varied as a parameter. Since the resonance frequency of the spin wave shifts with the application of *V*_G_, the frequency of the spin wave when 1.6 V was applied was considered to be the closest to the frequency of the input pulse signal, and resulted in excellent short-term memory and strong nonlinearity.


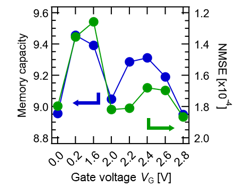


**Figure S15**. Memory capacity and NMSE variation at various *V*_G_ applications. The NMSE axis is inverted to compare the memory capacity and computational performance trends.

**S13. The number of nodes dependence of the memory capacity in the iono-magnonic reservoir**

The maximum step delay for the memory capacity evaluation was set to 20. It is set at such to avoid the integration of meaningless background, which does not contribute to improved performance. As shown in **Figure S16**a, the forgetting curve for 100 nodes asymptotically approaches *r*^2^(*τ*) = 0.0 at step delays above 20. On the other hand, as the number of nodes increases, forgetting becomes slower due to improvement of short-term memory performance. The *r*^2^(*τ*) does not reach 0.0, and a background with a finite value is observed. This background became more pronounced as the number of nodes increased. The step delay dependence of memory capacity, which is the integral of a forgetting curve, is shown in Figure S16b. The memory capacity when 100 nodes are used increased as the delay step increased from 1, and the increase slowed down and became nonlinear around a step delay of 6. The increase in memory capacity became negligibly small when the delay step was at or over 20. The range where the increase slowed down corresponds to the range where the *r*^2^(*τ*) shown in Figure S16a decreased significantly, and the *r*^2^(*τ*) approached a constant value near 0.0 at a step delay above 20, suggesting that it did not contribute to the increase in memory capacity. As the number of nodes increases, the slope in the linear region increases due to the large background in the forgetting curve, as shown in Figure S16a. Figure S16c summarizes the number of nodes dependence of step delay for the transition from nonlinear increase to linear increase in memory capacity. The step delay for the transition is 24 when 100 nodes are used, and the step delay increases with the number of nodes. This tendency results from improvement of the short-term memory property due to the increased number of nodes. Based on this step delay for the transition, we set the maximum step delay for the memory capacity calculation to 20 (indicated by arrows in Figure S16b and calculated the memory capacity for all conditions (100-800 nodes) in the range where the *r*^2^(*τ*) is meaningful. Particularly, in the range with a large number of nodes, relatively large *r*^2^(*τ*) remains as a background due to meaningless contribution, which in turn leads to meaningless increase in memory capacity. Thus, the calculations were performed so as not to include this background, and the calculated memory capacity was not overestimated.

The short-term memory capacity is also increased by increasing the dimension using *V*_G_, as shown in Figure S16d. The determination coefficient *r*^2^(τ) before the two steps increases as the number of nodes increases, due to the higher dimensionality of *V*_G_, which suggests an improvement in short-term memory performance. In addition, as shown in Figure S16e, the forgetting curves for 800 nodes show various behaviors for each interval. The *r*^2^(τ) for shorter (longer) intervals is larger when the delay step is τ ≤ 12 (12 ≤ τ). The short-term memory capacity can be calculated from the area of this forgetting curve.^[S1]^ Figure S16f shows the dependence of memory capacity on the number of nodes. As can be seen from the forgetting curves for different numbers of nodes, as shown in Figure S16d, the memory capacity increased as the number of nodes increased. As can be inferred from the forgetting curve shown in Figure S16e, there is no dependence on interval length in terms of memory capacity, and the highest memory capacity is achieved at an interval of 5 ns, which shows a memory capacity of 17.8. At 186 mT, on the other hand, the memory capacity increased monotonically with the number of nodes and the interval, as shown in Figure S16g. It was roughly equivalent to 169 mT up to an interval of 5 ns. However, at intervals exceeding 10 ns, the memory capacity rapidly increases, and the maximum memory capacity using 800 nodes was 19.7 at intervals of 15 ns and 20 ns. Since the benchmark tasks in this study (i.e., second-order nonlinear equation tasks and NARMA2 prediction tasks) are described by equations that depend on the state up to two steps before, as shown in Equations 5 and 6, these large memory capacities contribute to processing tasks with high accuracy.


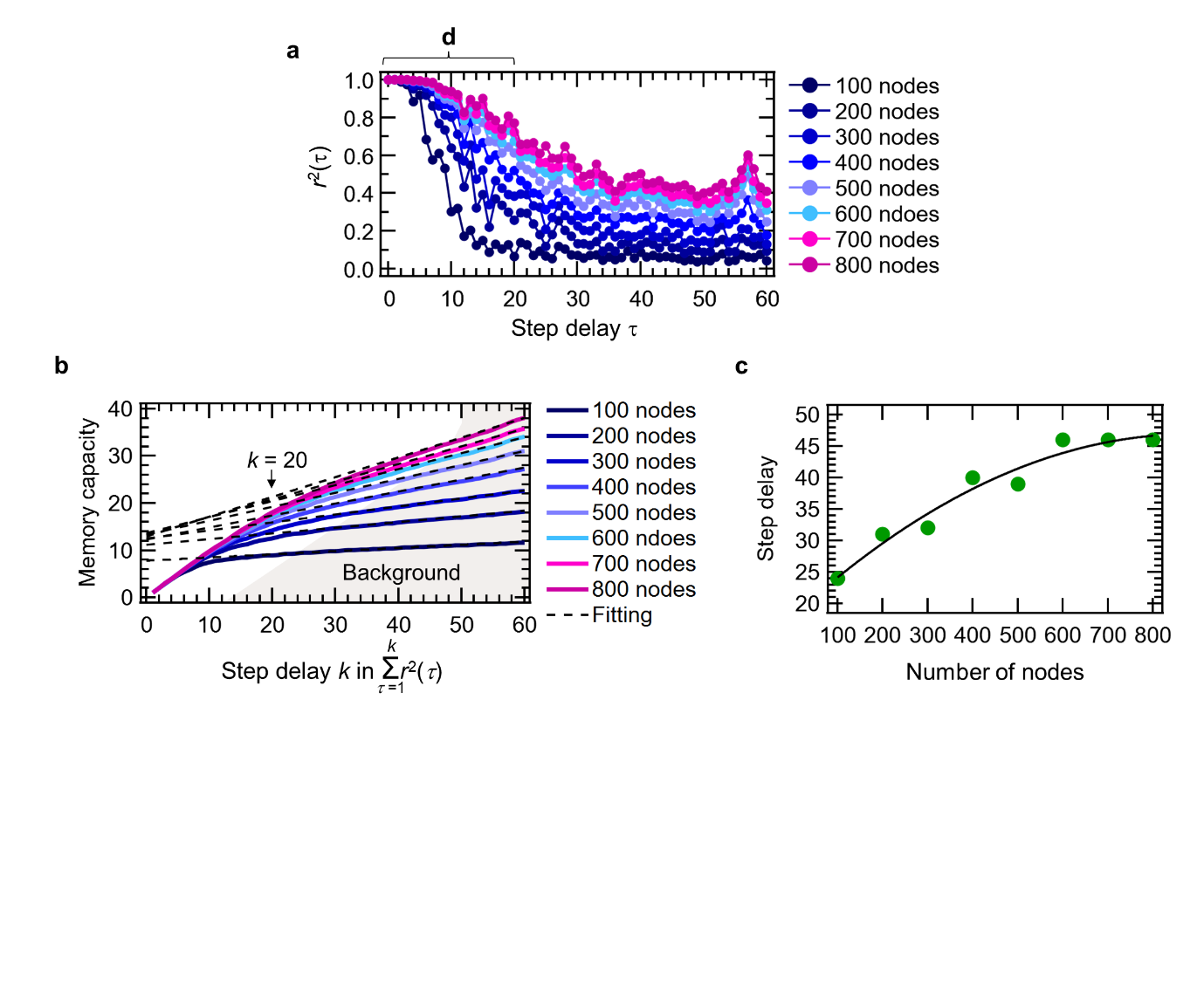

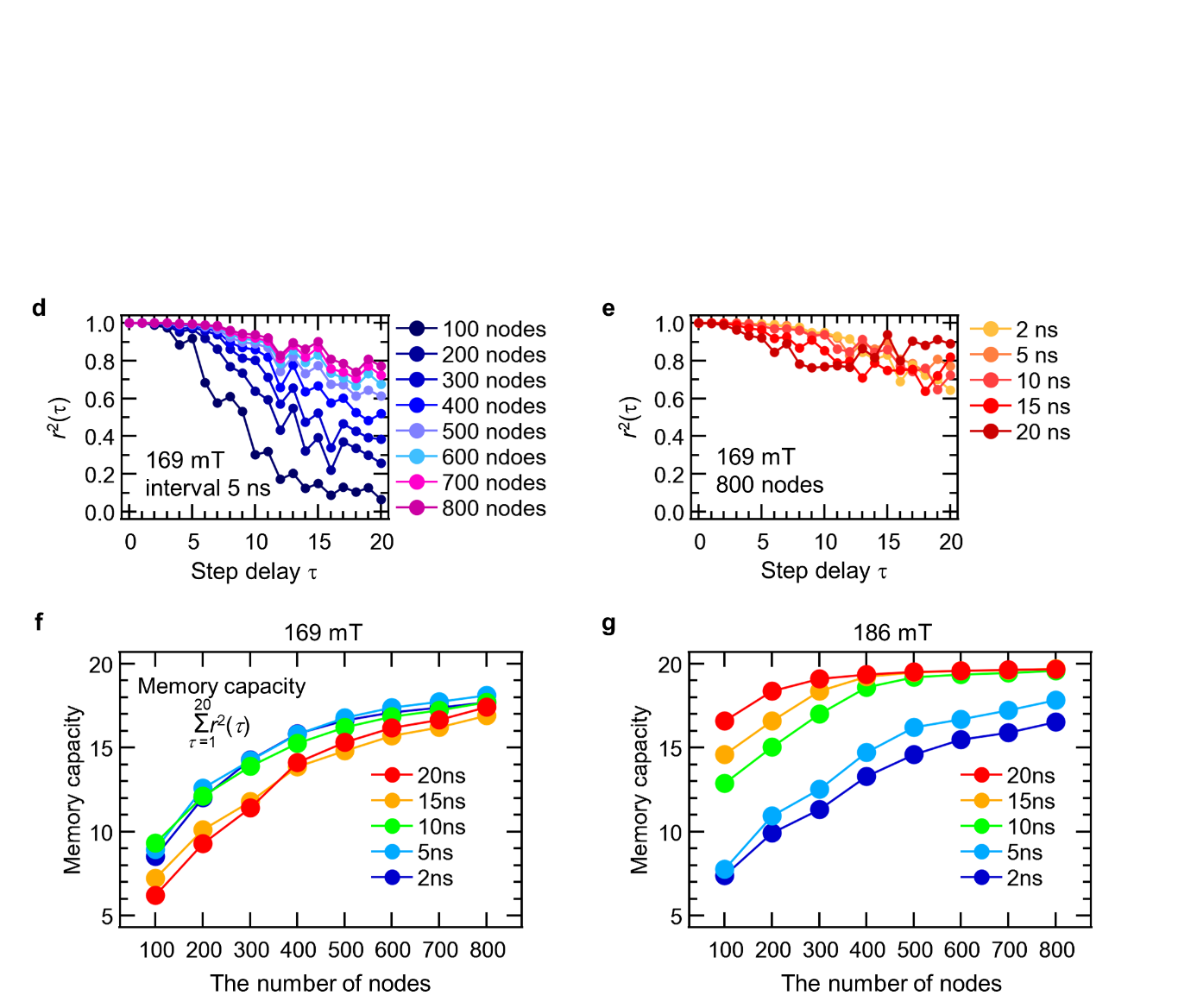


**Figure S16.** Evaluation of the short-term memory of the iono-magnonic reservoir, utilizing various spin wave properties induced by *V*_G_. a) Step delay τ dependence of the determination coefficient *r*^2^(τ) with a magnetic field of 169 mT and an interval of 5 ns, corresponding to the best condition for the second-order nonlinear equation and NARMA2 tasks. b) Memory capacity variation at various step delay *k*. Arrows denote the maximum step delay used to calculate the memory capacities shown in (f) and (g). The dashed lines are fitting results in the linear regions at each number of nodes. c) Number of nodes dependence of step delay for the transition from nonlinear increase to linear increase. d) τ dependence of *r*^2^(τ) at the cropped region of (a). e) τ dependence of *r*^2^(τ) in a magnetic field of 169 mT, with 800 nodes. f) Node number dependence of memory capacity of the iono-magnonic reservoir operating in a magnetic field of 169 mT. The memory capacity is determined as *r*^2^(τ) integrated at τ ranging from 1 to 20. g) Node number dependence of memory capacity of the iono-magnonic reservoir operating in a magnetic field of 186 mT.

**S14. Electric power consumption, operating speed, robustness of the iono-magnonic reservoir**

We showed good computational performance in this study. Here, we evaluated the subject iono-magnonic reservoir in terms of electric power consumption and operating speed. We calculated the power consumption of the iono-magnonic reservoir, which is attributed to the electric power for spin wave excitation and *V*_G_ application. The calculated power consumption is based on the electric power that the physical reservoirs consume per discrete time step, with such calculations used to compare it to other physical reservoirs. The electric power consumed by the iono-magnonic reservoir (*P*) is described as follows;

$P=A\left\{ \frac{1}{T}\int_{0}^{T} \frac{{V(t)}^{2}}{R}dt \right\}+\sum_{i=1}^{8} Q_{i}V_{G_{i}} (S5)$.

Here, *A*, *T*, *V*(*t*), *R*, *Q_i_*, and *V*_G_*_i_* are the accumulation number of 500, the total time step of 5000, input time of pulsed voltage used to excite a spin wave, a terminal resistance of 50 Ω, the migrated electric charge induced by *i*-th gate voltage during a period of 1800 seconds, and the applied *i*-th gate voltage. Its order is 0.0, 0.2, 1.6, 2.0, 2.2, 2.4, 2.6, and 2.8 V. The electric power consumed to excite spin waves under one *V*_G_ application and eight *V*_G_ applications are 114 nJ and 908 nJ, respectively. The electric power consumed by *V*_G_ application is 32.7 mJ. Thus, the *P* is approximately 32.7 mJ, which is the summation of 908 nJ and 32.7 mJ. We summarized the power consumption of physical reservoirs, as shown in **Table S1**. The electric power consumed in the iono-magnonic reservoir is large, compared to other physical reservoirs, due to long time for eight *V*_G_ applications. When the electric power consumed during *V*_G_ applications is eliminated, the electric power consumption is 908 nJ.

Table S1. A comparison of the power consumption.

| Types of Physical Reservoir Computing Devices | Consumption  /Step | Reference |
| --- | --- | --- |
| This study (including 1800 s *V*_G_ application) | 32.7 mJ |  |
| Few molecules | 201 µJ | [S16] |
| WO_x_-memristor | 143 µJ | [S17] |
| This study (excluding 1800 s *V*_G_ application) | 908 nJ |  |
| Spin wave interference | 114 nJ | [S1] |
| Ag-doped SiO_x_-memristor | 2.86 nJ | [S18] |
| TiO_x_/TaO_y_-memristor | 102 pJ | [S19] |
| Biomembrane-based memcapacitor | 41.5 fJ | [S20] |

Next, we calculated the operating speed of the iono-magnonic reservoir to compare it to other physical reservoirs. The operating times of the physical reservoir were calculated as follows^[S28]^: The number of steps required for training and testing by the physical reservoir in the NARMA task was multiplied by the input time per step. One waveform consisted of 5000 total steps (= discarded 500 steps/training phase of 3500 steps/discarded 500 steps/testing phase of 500 steps) and a 4 μs interval, where one step corresponds to the summation of the pulsed voltage and the interval between the pulsed voltage. Since this process is repeated 500 times for accumulation, all the time for operation (i.e., operating time) is described as follows,

Operating time = *A*(*T* × actual time per step + 4 μs) + 8*T*_V_. (S6)

Here, *T*_V_ is time to apply *V*_G_ (i.e., 1800 seconds). In order to compare the operating time for different physical reservoirs, we calculated it in the same way as above, using information found in the literature.^[S1,S7,S9,S14,S16,S17,S21-S28]^ **Figure S17** shows a benchmark of the operating time of the following physical reservoir devices; redox-ion gating reservoir (IGR),^[S8,S9]^ few molecule,^[S16]^ a random network of carbon nanotube,^[S21]^ micro electromechanical system,^[S22]^ Mackey-Glass oscillator,^[S14]^ WO_x_-memristor,^[S17]^ electric double layer (EDL)-IGR,^[S23]^ electrochemical cell,^[S7,S24]^ optical elements,^[S25-S27,S29]^ and spin wave interference with multi-detection.^[S1,S28]^ The operating time of the iono-magnonic reservoir, calculated by **equation S6**, is 14400 seconds. The time of voltage application is dominant, and the operating speed is slow compared to other physical reservoirs. In this operating time, the time required to input time-series data is 72 ms, and the time can be reduced to 9 ms, in correspondence with previous studies, by parallel processing with eight iono-magnonic reservoirs applying each voltage.


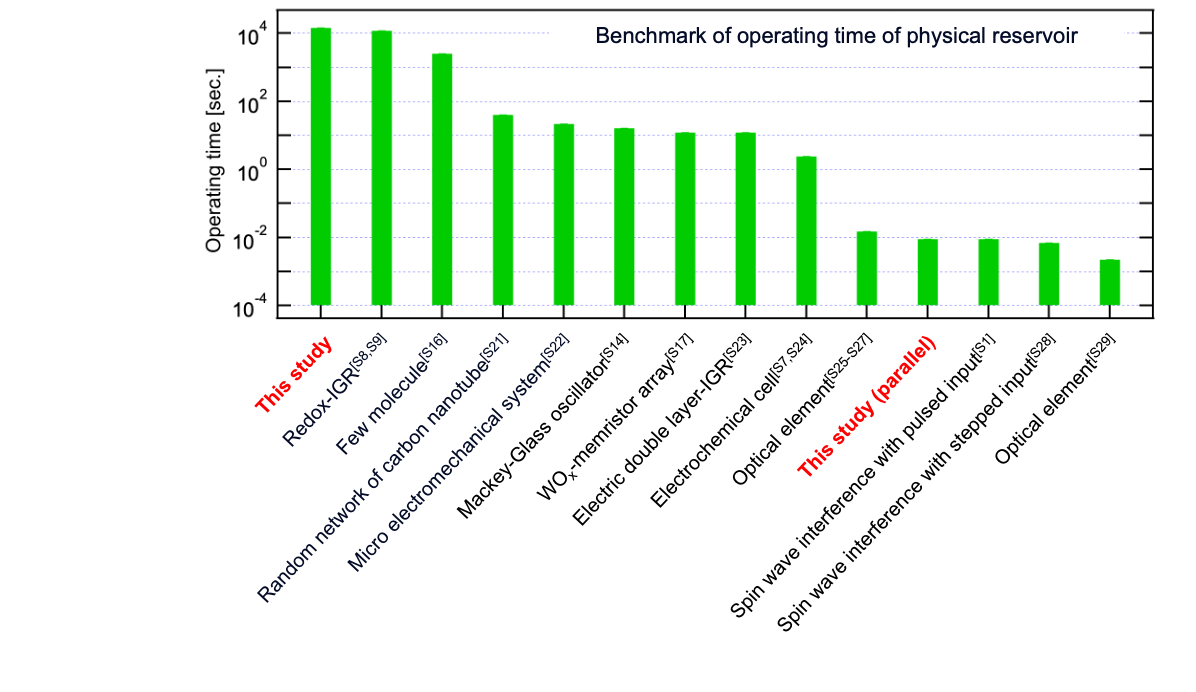


**Figure S17.** Benchmark of the operating time of various physical reservoir devices in experimental demonstrations. The operating time was calculated from the total time steps used to train and examine the trained reservoir network, including discarded time steps and time per step. Compared subjects are redox-IGR,^[S8,S9]^ few molecules,^[S16]^ random network of carbon nanotube,^[S21]^ micro electromechanical system,^[S22]^ Mackey-Glass oscillator,^[S14]^ WO_x_-memristor array,^[S17]^ electric double layer-IGR,^[S23]^ electrochemical cell,^[S7,S24]^ optical elements,^[S25-S27,S29]^ and spin wave interference with pulsed input and with stepped input.^[S1,S28]^

We evaluated the robustness of the reservoir network using a physical device. The reservoir network has generalized performance. Thus, the network with the weight parameter optimized in the training phase should exhibit processing performance, corresponding to performance at the training phase, for an input waveform that is not used in the training phase. We defined measurement of the robustness of the physical reservoir as *R*_r_ and calculated the ratio of NMSE for training and test phases, as follows;

$$R_{r}=\frac{1}{\left| \frac{\mathrm{NMSE}_{\mathrm{train}}}{\mathrm{NMSE}_{\mathrm{test}}}-1 \right|}, (S7)$$

where NMSE_train_ and NMSE_test_ are NMSEs at the training and test phases, respectively. The *R*_r_ increases as the robustness improves. To calculate the *R*_r_ in this work, NMSE_train_ and NMSE_test_ were set to 1.23×10^-4^ and 1.26×10^-4^, and the *R*_r_ is approximately 42. **Figure S18** shows summaries the *R*_r_ of phsyical reservoirs, which are optoelectronic system,^[S30]^ biomolecular memristor,^[S31]^ LiCoO_2_ redox-ion gating reservoir (IGR),^[S9]^ electric double layer (EDL)-IGR,^[S23]^ WO_x_-memristor,^[S17]^ spin torque oscillator,^[S32]^ nonlinear interfered spin wave multi-detection.^[S1]^. It was found that the iono-magnonic reservoir exhibits the strongest robusness, which in turn shows that the iono-magnonic reservoir has excellent generalized performance.


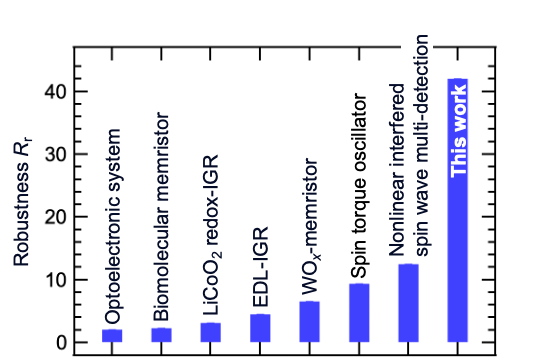


**Figure S18.** Robustness comparison among physical reservoirs. Optoelectronic system,^[S30]^ biomolecular memristor,^[S31]^ LiCoO_2_ redox-IGR,^[S9]^ EDL-IGR,^[S23]^ WO_x_-memristor,^[S17]^ spin torque oscillator,^[S32]^ nonlinear interfered spin wave multi-detection^[S1]^ are also shown.

**S15. Advantage of the ion-gating effect in the iono-magnonic reservoir**

The main advantage of the ion-gating effect for the physical reservoir is the ensuing ability to generate nonlinear and complex reservoir states in high dimensional space. Ion-gating can realize multiple spin waves with various properties, leading to diverse reservoir states. On the other hand, the drawbacks of the iono-magnonic reservoir are the required long operation times and high electric power consumption. However, these drawbacks can be overcome. Such long operating times in the iono-magnonic reservoir can be reduced by employing an array of eight devices. Eight combinations of two exciters, two detectors, and eight independent gate electrodes can be fabricated on the YIG. For reservoir computing, using these eight *V*_G_ states, in which voltage is applied to eight independent electrodes in advance, the waiting time of 14400 seconds to apply *V*_G_ can be reduced, which results in long computation times not being needed. This is because the eight combinations, consisting of a gate electrode and an antenna, operate independently and eliminate the need for waiting time when the *V*_G_ is changed. In addition, the eight reservoir states generated through each *V*_G_ state are acquired simultaneously, so eight measurements for eight reservoirs are no longer necessary, and the response of eight reservoirs can be acquired with a single measurement. By this contrivance, eight *V*_G_ states are realized in advance, and the time used for the calculation is only the time taken for the spin wave to respond to the input signal.

Furthermore, the high electric power consumption required can be also reduced by using parallel-connected devices. Each *V*_G_ state is realized in advance, so there is no need to change the gate voltage each time data is processed. Since the large current induced by the redox reaction at the interface between YIG and Nafion flows when changing the voltage, which migrates protons and changes the number of electronic carriers in the YIG, a method that does not change the voltage does not require a large current, and thus electric power in the system can be saved.

References

S1. W. Namiki, D. Nishioka, Y. Yamaguchi, T. Tsuchiya, T. Higuchi, K. Terabe, *Adv. Intell. Syst.* **2023**, *5*, 2300228.

S2. T. Goto, T. Yoshimoto, B. Iwamoto, K. Shimada, C. A. Ross, K. Sekiguchi, A. B. Graovsky, Y. Nakamura, H. Uchida, M. Inoue, *Sci. Rep.* **2019**, *9*, 16472.

S3. H. Ohno, D Chiba, F. Matsukawa, T. Omiya, E. Abe, T. Dietl, Y. Ohno, K. Otani, Nature **2000**, *408*, 944.

S4. M. Weisheit, S. Fahler, A. Marty, Y. Souche, C. Poinsingnon, D. Givord, Science **2007**, *315*, 349.

S5. A. Vansteenkiste, J. Leliaert, M. Dvornik, M. Helsen, F. Garcia-Sanchez, B. Van Waeyenberge, *AIP Adv.* **2014**, *4*, 107133.

S6. R. Nakane, G. Tanaka, A. Hirose, IEEE Access **2018**, 6, 4462.

S7. T. Shingu, H. Uchiyama, T. Watanabe, Y. Ohno, *Carbon* **2023**, *214*, 118344.

S8. T. Wada, D. Nishioka, W. Namiki, T. Tsuchiya, T. Higuchi, K. Terabe, *Adv. Intell. Syst.* **2023**, *5*, 2300123.

S9. K. Shibata, D. Nishioka, W. Namiki, T. Tsuchiya, T. Higuchi, K. Terabe, *Sci. Rep.* **2023**, *13*, 21060.

S10. K. Fukuda, Y. Horio, *Nonlinear Theory Appl. IEICE* **2021**, *12*, 639.

S11. R. López-Ruiz, H. L. Mancini, X. Calbe, *Phys. Lett. A* **1995**, *209*, 321.

S12. M. T. Martin, A. Plastino, O. A. Rosso, *Physica A* **2006**, *369*, 439.

S13. O. A. Rosso, H. A. Larrondo, M. T. Martin, A. Plastino, M. A. Fuentes, *Physl Rev. Lett.* **2007**, *99*, 154102.

S14. L. Appeltant, M. C. Sorano, G. van der Sande, J. Danckaert, S. Massar, J. Dambre, B. Schrauwen, C. R. Mirasso, I. Fischer, *Nat. Commun.* **2011**, *2*, 468.

S15. H. Jaeger, *German National Research Center for information Technology GMD Tech. Rep.* **2001**, *148*, 13.

S16. D. Nishioka, Y. Shingaya, T. Tsuchiya, T. Higuchi, K. Terabe, *Sci. Adv.* **2024**, *10*,

eadk6438.

S17. C. Du, F. Cai, M. A. Zidan, W. Ma, S. H. Lee, W. D. Lu, *Nat. Commun.* **2017**, *8*, 2204.

S18. R. Midya, Z. Wang, S. Asapu, X. Zhang, M. Rao, W. Song, Y. Zhuo, N. Upadhyay, J. J.

Yang, *Adv. Intell. Syst.* **2019**, *1*, 1900084.

S19. Y. Zhong, J. Tang, X. Li, B. Gao, H. Qian, H. Wu, *Nat. Commun.* **2021**, *12*, 408.

S20. M. R. Hossain, A. S. Mohamed, N. X. Armendarez, J. S. Najem, M. S. Hasan, *Adv.*

*Intell. Syst.* **2023**, *5*, 2300346.

S21. M. A. Kasaya, Y. Takeshima, S. Kan, K. Nakajima, T. Oya, T. Asai, *Neuromorph. Comput. Eng.* **2022**, *2*, 014003.

S22. B. Barazani, G. Dion, J.-F. Morissette, L. Beaudoin, J. Sylvestre, *J. Microelectromech. Syst.* **2020**, *29*, 338.

S23. D. Nishioka, T. Tsuchiya, W. Namiki, M. Takayanagi, M. Imura, Y. Koide, T. Higuchi, K. Terabe *Sci. Adv.* **2022**, *8*, eade1156.

S24. S. Kan, K. Nakajima, T. Asai, M. A. Kasaya, *Adv. Sci.* **2022**, *9*, 2104076.

S25. T. Okumura, M. Tai, M. Ando, *Nonlinear Theory Appl.* **2019**, *10*, 236.

S26. F. Duport, A. Smerieri, A. Akrout, M. Haelterman, S. Massar, *Sci. Rep.* **2016**, *6*, 22381.

S27. M. Hermans, P. Antonik, M. Haelterman, S. Massar, *Phys. Rev. Lett.* 2016, *117*, 128301.

S28. W. Namiki, D. Nishioka, T. Tsuchiya, K. Terabe, *Neuromorph. Comp. Eng.* **2024**, *4*, 024015.

S29. Q. Vinckier, F. Duport, A. Smerieri, K. Vandoorne, P. Bienstman, M. Haelterman, S. Massar, *Optica* **2015**, *2*, 438.

S30. J. Y. Sarrion, Master’s Thesis of Physics of Complex Systems at the University of Balearic

Island (2019).

S31. J. J. Maraj, K. P. T. Haughn, D. J. Inman, S. A. Sarles, *Adv. Intell. Syst.* **2023**, *5*, 2300049.

S32. W. Jiang, L. Chen, K. Zhou, L. Li, Q. Fu, Y. Du, R. H. Liu, *Appl. Phys. Lett.* **2019**, *115*,

192403.
